# An open-access T-BAS phylogeny for emerging *Phytophthora *species

**DOI:** 10.1371/journal.pone.0283540

**Published:** 2023-04-03

**Authors:** Allison Coomber, Amanda Saville, Ignazio Carbone, Jean Beagle Ristaino

**Affiliations:** 1 Department of Entomology and Plant Pathology, NC State University, Raleigh, North Carolina, United States of America; 2 Functional Genomics Program, NC State University, Raleigh, North Carolina, United States of America; 3 Center for Integrated Fungal Research, NC State University, Raleigh, North Carolina, United States of America; 4 Emerging Plant Disease and Global Food Security Cluster, NC State University, Raleigh, North Carolina, United States of America; Franklin & Marshall College, UNITED STATES

## Abstract

*Phytophthora* species cause severe diseases on food, forest, and ornamental crops. Since the genus was described in 1876, it has expanded to comprise over 190 formally described species. There is a need for an open access phylogenetic tool that centralizes diverse streams of sequence data and metadata to facilitate research and identification of *Phytophthora* species. We used the Tree-Based Alignment Selector Toolkit (T-BAS) to develop a phylogeny of 192 formally described species and 33 informal taxa in the genus *Phytophthora* using sequences of eight nuclear genes. The phylogenetic tree was inferred using the RAxML maximum likelihood program. A search engine was also developed to identify microsatellite genotypes of *P*. *infestans* based on genetic distance to known lineages. The T-BAS tool provides a visualization framework allowing users to place unknown isolates on a curated phylogeny of all *Phytophthora* species. Critically, the tree can be updated in real-time as new species are described. The tool contains metadata including clade, host species, substrate, sexual characteristics, distribution, and reference literature, which can be visualized on the tree and downloaded for other uses. This phylogenetic resource will allow data sharing among research groups and the database will enable the global *Phytophthora* community to upload sequences and determine the phylogenetic placement of an isolate within the larger phylogeny and to download sequence data and metadata. The database will be curated by a community of *Phytophthora* researchers and housed on the T-BAS web portal in the Center for Integrated Fungal Research at NC State. The T-BAS web tool can be leveraged to create similar metadata enhanced phylogenies for other Oomycete, bacterial or fungal pathogens.

## Introduction

*Phytophthora* is a genus of destructive, oomycete plant pathogens that cause devastating plant diseases on food crops, ornamentals, and in forest and riparian ecosystems [1]. *Phytophthora infestans* (Mont.) de Bary was the first species in the genus described, and was the causal agent responsible for the Irish potato famine in the 1840s [2, 3]. *Phytophthora infestans* causes late blight on tomato (*Solanum lycopersicum*) and potato (*Solanum tuberosum*) and remains an important threat to crop production globally [4, 5]. Other *Phytophthora* species also threaten agricultural production systems and natural ecosystems [1, 6, 7]. Important examples include *P*. *ramorum*, responsible for the disease known as sudden oak death and *P*. *cinnamomi*, a generalist species with a wide host range [7–10]. In western Africa, *P*. *megakarya* is responsible for black pod disease of cacao and threatens cacao production and *P*. *palmivora* threatens cacao production globally [11]. Globally, other *Phytophthora* species cause destructive plant diseases in many other plant species [1, 12].

**Fig 1:**
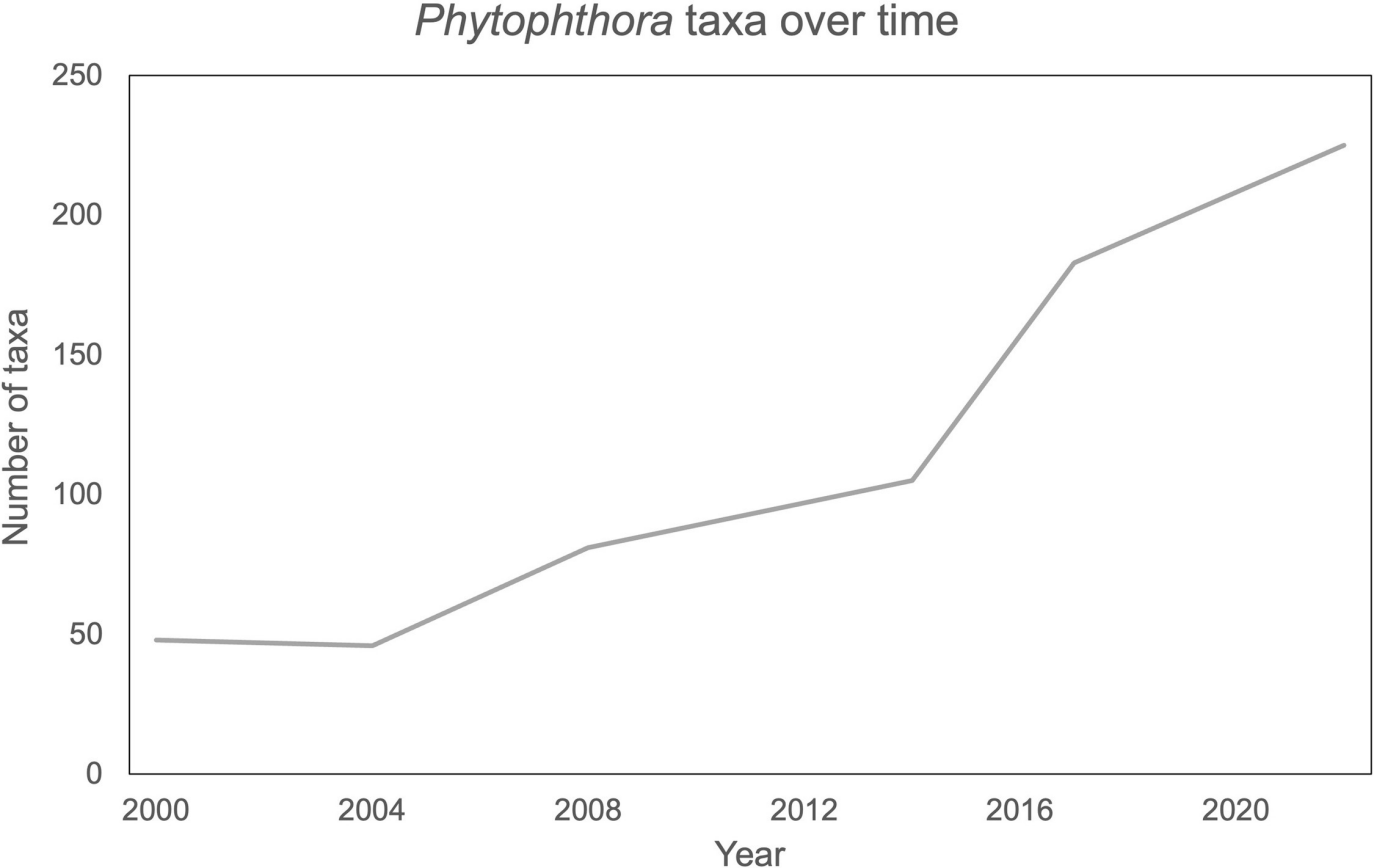
Number of species in the *Phytophthora* genus. Line graph showing the number of *Phytophthora* species included in different phylogenies from 2000 to the current study.

Diverse new species of *Phytophthora* are regularly being described from croplands, forests, and water ecosystems around the world [6, 13, 14]. To date, more than 200 species have been formally described, most of them in the last 15 years [1, 6, 14]. More species are expected to be discovered or described as surveys of water, riparian forest buffer plants, and forest ecosystems continue [15, 17]. There has been an exponential expansion in the number of new species described in recent years (Fig 1).

Historically, morphological characteristics have been used to identify *Phytophthora* species [15–23]. Morphological groupings and dichotomous keys have been useful tools in identifying *Phytophthora* species based on observed morphological characteristics [17, 18, 20–22]. A matrix-based Lucid Key has also been developed for identification of common *Phytophthora* species [19]. This resource incorporated both morphological and molecular characteristics (*ITS* and *CoxI* genes) to aid in species identification but only represented 50 common species [19]. However, in the past 10 years, the number of new species discovered has expanded greatly, resulting in many recently described species that are not included in the Lucid key resource.

Species identification has relied more on molecular identification methods such as single gene or multilocus sequencing and whole genome sequencing [17, 24]. This has also enabled the production of more robust phylogenies based on sequence similarity [17, 25, 26]. In 2000, a phylogeny of 50 *Phytophthora* species was developed by Cooke and colleagues based on *ITS* sequence data [25]. Since then, expanded phylogenies with additional species and loci have been developed as both the genus and sequencing resources have grown [14, 26–31]. Yang and colleagues presented a robust phylogeny for the genus, including many newly described species and isolates that have since been described as individual species [14]. They and many others have used multilocus genotyping in *Phytophthora* to differentiate species [14, 32].

Although multilocus sequencing generates robust phylogenies, the morphological and biological information of the associated species is still of high importance to researchers and is often left out of resulting phylogenies. The recent IDphy tool developed by Abad et al. (2022) is an exception and includes a detailed Lucid key for species identification using morphology of over 160 species, detailed fact sheets with images and tools for running BLAST`searches at NCBI to identify species using multiple locus sequences [16]. This work also stressed the importance of well-curated type species in phylogenies.

The current phylogenetic system and curated knowledge on the *Phytophthora* genus is disjointed, with molecular phylogenies and biological information being presented in disparate resources. Several databases with biological and sequence information on *Phytophthora* are available, but those systems do not integrate new biological information from newly described species, nor do they allow for expandable living phylogenies to be created and curated by the research community [33–35]. There is a need to connect available *Phytophthora* multilocus sequence data into a centralized maximum likelihood tree with metadata to facilitate species identification and to study the evolution and emergence of species within the genus.

The software toolkit “T-BAS” has been developed for the integration of phylogenetic placement and visualization of biological metadata [36, 37]. This NSF-funded resource has been used effectively for development and presentation of fungal phylogenies, especially for the Ascomycota [38, 39]. The effectiveness of T-BAS for use in other fungal systems, including plant pathogens, indicates its potential utility for making a more centralized resource for *Phytophthora* identification and phylogenetic data. We harnessed the T-BAS system to build a living phylogeny of *Phytophthora*, incorporating sequence data and metadata for most of the recently described species. Most importantly, the live phylogeny format allows for the rapid, curated placement of new species and taxa as they are described.

Differentiation of genotypes within a species of *Phytophthora* is also important. *Phytophthora infestans* consists of multiple clonal lineages that scientists differentiate to inform research and management practices [40]. *Phytophthora infestans* populations are dominated by clonal lineages that have different phenotypes, such as fungicide sensitivity and host preference [41]. Tracking the spread and prevalence of these clonal lineages is critical to late blight management in both the United States and Europe [4]. The temporal and geographic distribution of lineages is monitored by researchers on USABlight and EuroBlight in these two regions, respectively [4, 40, 42, 43]. The current standard practice to identify a lineage of *P*. *infestans* involves amplification of 12 microsatellite (SSR) markers [40, 42, 44]. Once the markers are amplified, they are typed according to the number and size of alleles at the 12 loci using the protocol outlined by Li and colleagues [44]. The system in place for identification of *P*. *infestans* lineages relies on the expertise of a few well-trained researchers and there is no centralized queryable database of genotypes. One online tool, Phytophthora-ID: Genotype-ID has been developed, but not all recent global genotypes are incorporated into that database [34]. Like species identification within the genus, *Phytophthora infestans* lineage identification would benefit from a centralized open resource with molecular and biological data integrated and curated by the late blight research community.

Given the disparate datasets and expansion in reports on new species of *Phytophthora*, a more centralized system for curating sequence data and inferring robust phylogenies is needed [15]. The primary objectives of this work were to: 1) Develop an open T-BAS phylogeny for *Phytophthora* by synthesizing multiocus sequence data, biological trait data, and metadata from various published sources; 2) Make this phylogeny comprehensive and easily updatable by the research community to keep pace with discovery of new *Phytophthora* species; 3) Develop a queryable search engine for *P*. *infestans* SSR data for the identification of genotypes.

## Methods

### Sequence data collection

Publicly available sequence data for described *Phytophthora* species were downloaded for nine loci (*28S*, *60SL10*, *Btub*, *EF1α*, *Enl*, *HSP90*, *TigA*, *ITS*, and *CoxI*) from GenBank, drawing on previous phylogenetic works, species descriptions for new species, and the molecular key developed as part of IDPhy (S1 Table) [14]. All of these are nuclear loci with the exception of the *CoxI* mitochondrial locus.

Unfortunately, many *Phytophthora* isolates in GenBank have either been misidentified or renamed as more studies are done [16]. We used *ITS* and *CoxI* datasets from the well- researched IDPhy types or extype collections where available. For the other loci, we drew on well-supported sequence data from prior phylogenies of the genus. For the newer species not included in previous phylogenies or IDPhy, we used sequence data from species description papers. We also reviewed the isolates used for taxa that have known misidentifications like *P*. *richardiae* to ensure we used sequence data from the correct species. By comparing our cultures and notes, the culture collection and notes of Mannon Gallegly, the fact sheets provided on IDPhy, and recent species descriptions we were able to synthesize a thorough and accurate dataset of *Phytophthora* sequences.

Building on this dataset, we sequenced seven of these loci (*28S*, *60SL10*, *Btub*, *EF1α*, *Enl*, *HSP90*, and *TigA*) for two additional species of *Phytophthora* which were recently described, *Phytophthora acaciae* and *Phytophthora betacei* (S1 Table) [45, 46]. *Phytophthora acaciae* was described as a species in Brazil in 2019 and infects black wattle (*Acacia mearnsii*) [45]. *Phytophthora betacei* was described in Colombia in 2018 and is a pathogen of tree tomato (*Solanum betaceum*), an important crop species in that area [46]. This species was previously classified as *P*. *andina* lineage EC-3 in earlier published work [47, 48] and is of interest to us in a related project on phylogenetic placement of this species. We also tested placement of *P*. *cryptogea*, *P*. *ramorum* and *P*. *nicotianae* in the tree using multilocus sequence datasets generated in our lab.

Isolates of *P*. *acaciae* were obtained from Dauri Tessmen, Universidade Estadual de Maringá, Parana, Brazil. Isolates of *P*. *betacei* were obtained from Sylvia Restrepo, University of Los Andes, Colombia. DNA extraction was performed using the CTAB method as described previously [49]. The primers used to amplify each of the seven loci were from previously published works (S2 Table) [14, 25, 27, 28, 32, 50]. PCR reactions were done in 50 μL volumes. Each 50 μL reaction contained 5 μL of 10X PCR buffer (Genesee, SanDiego, CA), 2.5 μL dNTPs (2 mM per nucleotide), 2 μL each 10 μM forward and reverse primer, 1.8 μL MgCl_2_ (50mg/mL), 0.25 μL BSA (20mg/mL), 0.2 μL Taq (5U/μL) (Genesee, SanDiego, CA), with the remainder to 49 μL as dd H_2_O. The final 1 μL consisted of sample DNA. Thermal cycling protocol consisted of 94°C for 5 minutes; then cycles of 94°C for 2 minutes, an annealing step specific to the primers, and 72°C for 2 minutes; followed by a final extension period at 72°C for 2 minutes (S2 Table). For the primers amplifying the TigA and HS90 loci, the annealing step was for 30 seconds with temperatures ranging from 64°C to 62°C, and 12 cycles completed at each temperature. For the other regions, the annealing step was for 30 seconds with temperatures ranging from 60°C to 53°C, and 3 cycles completed at each temperature plus an additional fifteen cycles with an annealing temperature of 53°C. Temperature reductions in the annealing step over time, known as touchdown PCR, allowed for amplification of multiple loci in one thermocycler protocol. Detailed descriptions of the primers, their optimum annealing temperatures, and the sources they were adapted from can be found in S2 Table. Amplicons from the PCR reactions that were expected to contain the locus of interest were sequenced using Sanger sequencing at the Genomic Sciences Laboratory at North Carolina State University.

In total, sequence data was collected for 194 *Phytophthora* species, 30 informally described *Phytophthora* taxa, and 3 outgroups from related oomycete genera (S1 Table). Compared to the most recent phylogeny using this larger set of loci, this represents the addition of 50 new taxa [14].

### Phylogenetic inference and visualization

Phylogenetic trees were inferred with 1000 bootstrap replicates under the GTRGAMMA model using RAxML version 8 via the CIPRES REST API implemented in the DeCIFR toolkit (https://tools.decifr.hpc.ncsu.edu/denovo) [51, 52]. We partitioned the data by locus for our phylogeny. We also rebuilt the tree using IQTREE with partitioning by codon as well as separate partitions for the third codon positions.

In total, three sets of phylogenetic trees were generated. First, a phylogenetic tree based on the eight nuclear loci was constructed after excluding taxa with sequence data for fewer than three loci. An additional phylogenetic tree consisting of only the mitochondrial *CoxI* locus was inferred for species that had sequence data available at this locus. Lastly, independent trees for each locus were also inferred separately. Trees for all nine loci were compared using the Hypha package module of Mesquite v3.51 implemented in the DeCIFR toolkit (https://tools.decifr.hpc.ncsu.edu/trees2hypha) [53, 54]. The tree based on the eight nuclear loci was selected for further analysis.

The resulting *Phytophthora* genus tree was uploaded into the Tree-Based Alignment Selector Toolkit (T-BAS version 2.3) developed by Ignazio Carbone and colleagues [36]. The T-BAS system was chosen because it has a phylogeny-based placement feature that allows incorporation of new taxa into an existing phylogenetic tree. Metadata for the taxa in the tree were collected using a custom Python script for web scraping with the package BeautifulSoup as well as manual data collection [55]. A portion of the metadata for some species was retrieved from IDPhy, a published, open-access *Phytophthora* database developed and curated by Gloria Abad and colleagues [16, 33]. Metadata for some traits was summarized into a smaller number of categories to facilitate visualization. For example, host range was characterized as ‘specific’ or ‘broad’ following the categories presented in [8]. Each species was manually encoded to fit into one of these categories based on the number of host plants and their relatedness. *Phytophthora* species with few closely related hosts were placed in the specific category; hosts in three or more plant families were considered broad. For species where host information was lacking, a no host described category was used. A full list of specific hosts was also retained in the metadata. Similarly, pathogen lifestyle was categorized into soilborne, aquatic, and/or aerial [33]. Above ground disease symptoms were categorized as aerial, while below ground root infections were categorized as soilborne. Specific pathogen lifestyle information was also retained in the metadata. *Phytophthora* species with unknown lifestyles were left blank. Sexual characteristics were encoded as homothallic (ie, self-fertile) or heterothallic (ie, outcrossing), where available, or coded as sterile using the definition of Brasier et al. [15]. All distribution data were both retained in full and summarized into a list of continents for each species. The visualization capabilities of T-BAS were used to add metadata such as clade, sexual characteristics, or lifestyle traits to the phylogenetic tree inferred from the sequence data. Users have the option to download single and multilocus DNA sequence alignments, metadata, and Newick-formatted trees for focal clades of interest.

### Phylogeny validation

To test real-time phylogenetic placement onto the tree, sequence data for two newly described *Phytophthora* species sequenced in this study (*P*. *betacei and P acaciae*) were added to the phylogeny. We used *P*. *betacei* because it is a close sister species to *P*. *andina* but has a different mitochondrial haplotype and is of interest to us in related sequencing project. *Phytophthora andina* has a 1c mitochondrial haplotype and *P*. *betacei* is 1a.The new taxa were inserted in the tree using the phylogeny-based evolutionary placement algorithm (EPA) implemented in T-BAS [36, 56, 57]. Additionally, independently identified species of *Phytophthora* from nursery crop plants including *P*. *nicotianae*, *P*. *tropicalis*, *P*. *palmivora*, *P*. *drechsleri*, *P*. *cryptogea*, *P*. *pseudocryptogea*, *and P*. *kelmania* collected by graduate student and extension specialist Inga Meadows and species that infect rhododendron were placed in the multilocus tree using the *Phytophthora* reference tree as a backbone constraint tree and 1,000 bootstrap replicates in RAxML. A tutorial and practice FASTA files for placement of two “unknown” *Phytophthora* species can be found on the project GitHub repository (https://github.com/allisoncoomber/phytophthora_tbas) and in S1 File.

### *P*. *infestans* microsatellite lineage classifier

A dataset of approximately 2200 *Phytophthora infestans* isolates of known lineages and their corresponding microsatellite genotypes were obtained from over thirty years of collecting by Jean Beagle Ristaino [4, 43, 58]. Using R, an applet was developed to compare newly typed isolates of *P*. *infestans* to this dataset of lineages [59]. For genotype identification, unknown isolates were compared to a reference genotype using Bruvo’s genetic distance algorithm, implemented by the R package Poppr [60, 61]. Bruvo’s distance was selected for comparing unknown genotypes to the reference because it compares the relative genetic closeness of samples regardless of the ploidy [60]. *Phytophthora infestans* has been documented to have variations in ploidy from diploid to tetraploid, with triploid clonal lineages being common [62]. Variations in ploidy can influence results of other common genetic distance methods, so Bruvo’s distance is a logical choice for this species [60]. A newly typed isolate was considered to belong to a genotype if it was within a threshold genetic distance of other isolates in that genotype (described below).

A user interface was developed for the applet using the R Shiny package. The SSR genotype classifier is linked to the USABlight.org website under the “Identify an SSR genotype” page. A tutorial describing how to use the classifier and example files can be found in S2 File.

A threshold genetic distance to differentiate lineages was estimated using the cutoff-predictor function from Poppr [61]. A histogram of Bruvo’s values for all pairwise comparisons also provided visual confirmation of the threshold value (S1 Fig). Biologically, this threshold distance approximates the distinction between clonally and sexually related pairs, allowing the classifier to distinguish clonal genotypes.

The applet was also tested using a minimum representative set of distinct reference genotypes representing global populations of *P*. *infestans* to investigate which genotypes were similar and likely to be confused for each other. In this case, the reference set was developed by selecting the most common microsatellite profile for each genotype in the dataset. The remainder of isolates were then used as the tester dataset. These parameters produced a similar cutoff threshold to that estimated by Poppr.

To test the sensitivity and specificity of the classifier, the reference dataset was split into five randomly selected, non-overlapping samples. Each sample was classified using the remainder of the reference dataset (i.e., those not included in the sample). For some lineages which had only one or a few representative isolates, sensitivity and specificity could not be estimated.

## Results

### Live phylogeny of *Phytophthora*

**Fig 2:**
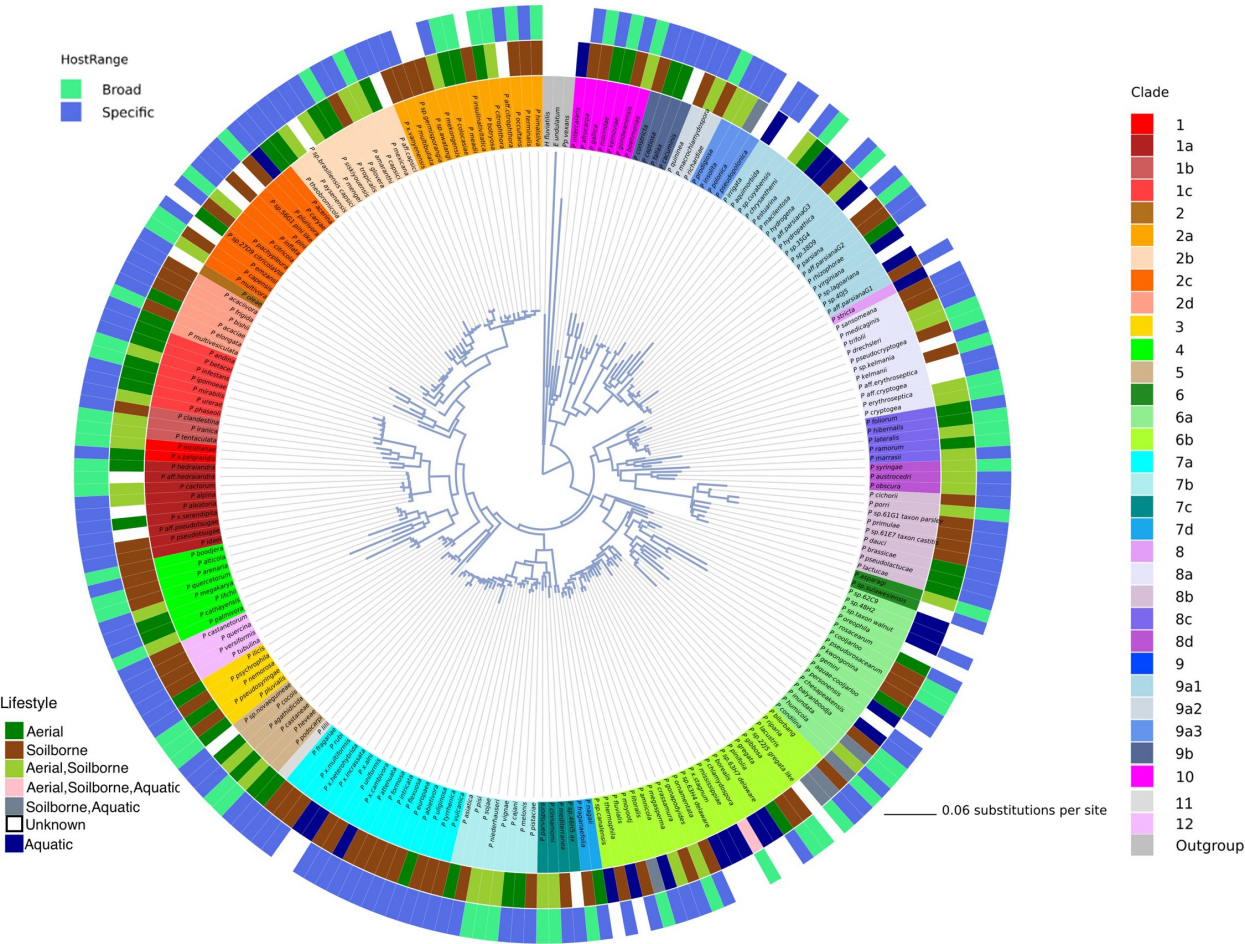
Phylogeny of the *Phytophthora* genus. Radial phylogeny of the genus *Phytophthora* inferred with maximum likelihood and 1,000 bootstrap replicates for an alignment of 8 concatenated nuclear genes. Coloring on the inner ring indicates clade. Colors on the middle ring indicate substrate. Colors on the outer ring indicate host range (broad, specific, or no host reported). The branches in the tree are drawn to scale and the scale bar represents 0.06 substitutions per site.

A total of 194 formally described *Phytophthora* species and 33 affiliated taxa were included in the genus phylogeny (Fig 2 and S1 Table). The maximum likelihood phylogeny was inferred with RAxML and includes 8 concatenated nuclear loci (*28S*, *60SL10*, *Btub*, *EF1α*, *ENL*, *HS90*, *ITS*, and *TigA*). The tree was rooted with three outgroups from related oomycete genera (*Halophytophthora fluviatilis*, *Phytopythium vexans*, and *Elongisporangium undulatum*) (Fig 2). A full list of included species and accession numbers for each locus can be found in S1 Table. There was strong support for the outgroups as separate and distinct from the rest of the *Phytophthora* genus, with the *Phytophthora* genus appearing as a monophyletic clade (Fig 2). Note no downy mildew pathogen sequences were used in our phylogeny as our focus was on *Phytophthora*, but the genus *Phytophthora* is known to be paraphyletic with downy mildews residing within provisional clades as outlined by Bourret et al (2018) [15, 63].

**Fig 3:**
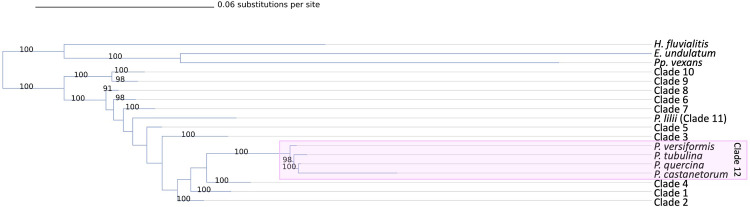
Phylogeny of major *Phytophthora* clades. Collapsed phylogenetic tree of the genus *Phytophthora* showing the relationships between the clades, outgroups, and species that do not fit into the conventional clade system. Clade 12 is highlighted in pink. Phylogeny is inferred using maximum likelihood and 1,000 bootstrap replicates for an alignment of 8 concatenated nuclear loci. Bootstrap values (in percent) are shown above each branch. The branches in the tree are drawn to scale and the scale bar represents 0.06 substitutions per site.

The results of this phylogeny were largely in agreement with what has been previously presented [14, 25–27, 29–31]. There was strong evidence for twelve subclades within the genus, and the relationships between clades were mostly in agreement with previous studies (Fig 3). Clade 11 contained one species, *P*. *lilii*, and clade 12 contains *P*. *castenatorum*, *P quercina*, *P*. *tubulina* and *P*. *versiformis*. Fifty species or closely affiliated taxa were placed in our tree that were not reported in the previous phylogeny by Yang et al. (2017) (Table 1 and S2 Fig). Placement of these taxa was overall in agreement with their previous descriptions (references for each in Table 1).

**Table 1:** Recently described *Phytophthora* taxa in TBAS phylogeny along with a summary of their associated metadata and species description paper.

Species	Previous Clade	Current Clade	Sexual Characteristic	Host Species	Substrate	Location	Source
*P*. *abietivora*	-	7a	Sterile	*Abies fraseri*	stems	US: CT	[64]
*P*. *acaciae*	2	2d	Heterothallic	*Acacia mearnsii*	bark tissue	Brazil	[45]
*P*. *acaciivora*	-	2d	Heterothallic	*Acacia mangiuma*	roots	Vietnam	[65]
*P*. *afrocarpa*	-	10	Sterile	*Afrocarpus falcatus*	rhizosphere	South Africa	[66]
*P*. *aleatoria*	1	1a	Homothallic	*Pinus radiata*	bark, stems, branches	New Zealand	[67]
*P*. *alpina*	-	1a	Homothallic	*Alnus viridis*	rhizosphere, bleeding cankers	Italy	[68]
*P*. *amaranthi*	-	2b	Homothallic	*Amaranthus tricolor*, *A*. *viridis*	leaves, roots, and stems of inoculated plants	Taiwan	[69]
*P*. *aquae-cooljarloo*	-	6a	Homothallic	Unknown	pond water	Australia	[70]
*P*. *aysenensis*	2	2b	Homothallic	*Aristotelia chilensis*	collar, stem, rhizosphere	Chile	[71]
*P*. *balyanboodja*	-	6a	Sterile	Unknown	rhizosphere	Australia	[72]
*P*. *betacei*	-	1c	Heterothallic	*Solanum betaceum*	leaves	Colombia	[46]
*P*. *boodjera*	-	4	Homothallic	*Agonis flexuosa*, *Eucalyptus spp*., *Xanthorrhoea preissii*, *Corymbia calophylla*	soil and root baits	Australia	[73]
*P*. *cacuminis*	9	9b	Sterile	Unknown	Unknown	Australia	[74]
*P*. *caryae*	-	2c	Homothallic	*Carya*	water	US: MA, NC	[75]
*P*. *castanetorum*	3b	12	Homothallic	*Castanea sativa*	rhizosphere	Italy; Portugal	[13]
*P*. *cathayensis*	-	4	Homothallic	*Carya cathayensis*	cambium collar canker	southeast China	[76]
*P*. *chesapeakensis*	6	6a	Sterile	*Zostera marina*	seeds	Chesapeake Bay	[77]
*P*. *chlamydospora*	-	6b	Sterile	Unknown	roots, leaves, water, soil	Unknown	[78]
*P*. *condilina*	-	6a	Homothallic	*Casuarina obesa*	rhizosphere	Australia	[72]
*P*. *cooljarloo*	-	6a	Homothallic	*Hibberia sp*.	rhizosphere	Australia	[72]
*P*. *estuarina*	9	9a1	Sterile	*Laguncularia racemose*, *Sorghum sp*., *Rhizophora mangle*	leaves, seeds	Brazil	[79]
*P*. *insulinativitatica*	-	2a	Heterothallic	Unknown	disturbed rainforest rhizosphere	Australia, Christmas Island	[80]
*P*. *kelmanii*	-	8a	Heterothallic	*Ptilotus pyramidatus*, *Xanthorrhea pressii*, *Salvia rosmarinus*, *Juglans nigra*	rhizosphere	Australia; US: CA	[81]
*P*. *kwongonina*	-	6a	Homothallic	*Banksia grandis*	rhizosphere	Western Australia	[72]
*P*. *litchii*	-	4	Homothallic	*Litchi chinensis*	fruits	Taiwan, Netherlands	[82]
*P*. *marrasii*	-	8c	Sterile	*Cynara cardunculus*	crown and root	Italy	[83]
*P*. *mediterranea*	-	7c	Heterothallic	*Myrtus communis*	roots	Italy	[84]
*P*. *mekongensis*	-	2a	Sterile	*Citrus grandis*	roots, fruits	Vietnam	[85]
*P*. *moyootj*	-	6b	Sterile	Unknown	soil	Australia	[86]
*P*. *multibullata*	-	2a	Heterothallic	*Cinnamomum cassia*	rhizosphere	Vietnam	[80]
*P*. *oleae*	-	2	Homothallic	Unknown	Unknown	Unknown	[87]
*P*. *oreophila*	-	6a	Homothallic	Unknown	Unknown	Unknown	[74]
*P*. *podocarpi*	unknown	5	Homothallic	*Podocarpus totara*	needles	New Zealand	[88]
*P*. *prodigiosa*	9	9a3	Sterile	*Citrus grandis*	roots, fruits	Vietnam	[85]
*P*. *pseudolactucae*	-	8b	Homothallic	*Lactuca sativa*	stem, crown	Japan	[89]
*P*. *pseudopolonica*	9	9a3	Homothallic	Unknown	Unknown	China	[90]
*P*. *pseudorosacearum*	-	6a	Homothallic	*Xanthorrhea platyphylla*, *Persoonia longifolia*	rhizosphere	Australia	[72]
*P*. *rhizophorae*	9	9a1	Sterile	*Rhizophora mangle*, *Sorghum sp*.	leaves, seeds	Brazil	[79]
*P*. *theobromicola*	2	2b	Sterile	*Theobroma cacao*	pods	Brazil, Bahia: Eunápolis	[91]
*P*. *tubulina*	3b	12	Homothallic	*Fagus sylvatica*	rhizosphere	Austria	[13]
*P*. *tyrrhenica*	-	7a	Homothallic	*Quercus ilex*, *Q*. *suber*	rhizosphere	Italy	[13]
*P*. *urerae*	-	1c	Heterothallic	*Urera laciniata*	leaves	Peru	[92]
*P*. *versiformis*	3b	12	Homothallic	*Corymbia calophylla*	rhizosphere	Australia	[93]
*P*. *vulcanica*	-	7a	Homothallic	*Fagus sylvatica*	rhizosphere	Italy	[13]
*P*. sp. awatangi^**1**^	-	2a	Heterothallic	Unknown	disturbed rainforest rhizosphere	Papua New Guinea	[80]
*P*. sp. germisporangia^**1**^	-	2a	Heterothallic	Unknown	disturbed rainforest rhizosphere	Papua New Guinea	[80]
*P*. sp. novaeguineae^**1**^	-	5	Sterile	Unknown	Unknown	Unknown	M. Coffey, unpublished
*P*. x *pelgrandis*^*2*^	unknown	1	Homothallic	*Pelargonium grandiflorum*	stalks	Taiwan; Germany	[94]
*P*. x *serendipita*^*2*^	unknown	1a	Homothallic	*Idesia polycarpa*	stem base	Netherlands	[95]
*P*. x *vanyenensis*^*2*^	-	2a	Heterothallic	*Cinnamomum cassia*	rhizosphere	Vietnam	[80]

^1^Not a formally described species. Referred to here with informal and/or isolate name.

^2^Hybrid

Several taxa that were previously given only a general clade in their species description papers were resolved to the subclade level by our study, similar to Chen et al.’s (2021) recent phylogeny (Table 1). For example, *P*. *prodigiosa*, *P*. *pseudopolonica*, *P*. *rhizophorae*, *P*. *estuarina*, and *P*. *cacuminis* were all previously described as belonging to clade 9 in their species description papers. Here, we resolve them to the subclade or subclade cluster level as 9a3, 9a3, 9a1, 9a1, and 9b respectively (S3 Fig), matching the results of Chen et al. 2021. Additional subclade distinctions and their previous clade classifications are listed in Table 1. Notably, the addition of several new species closely related to *P*. *quercina* including *P*. *versiformis*, *P*. *tubulina*, and *P*. *castanetorum*, thus allowing for a more detailed resolution of clade 12 (Fig 3). This clade was previously referred to as 3b [16, 33].

The topology of the *CoxI* mitochondrial tree differed from the nuclear derived trees, (S3 and S4 Figs). Because of this discordance, the *CoxI* locus was included as a single locus tree, but not with the other nuclear loci in the multilocus tree.

**Fig 4:**
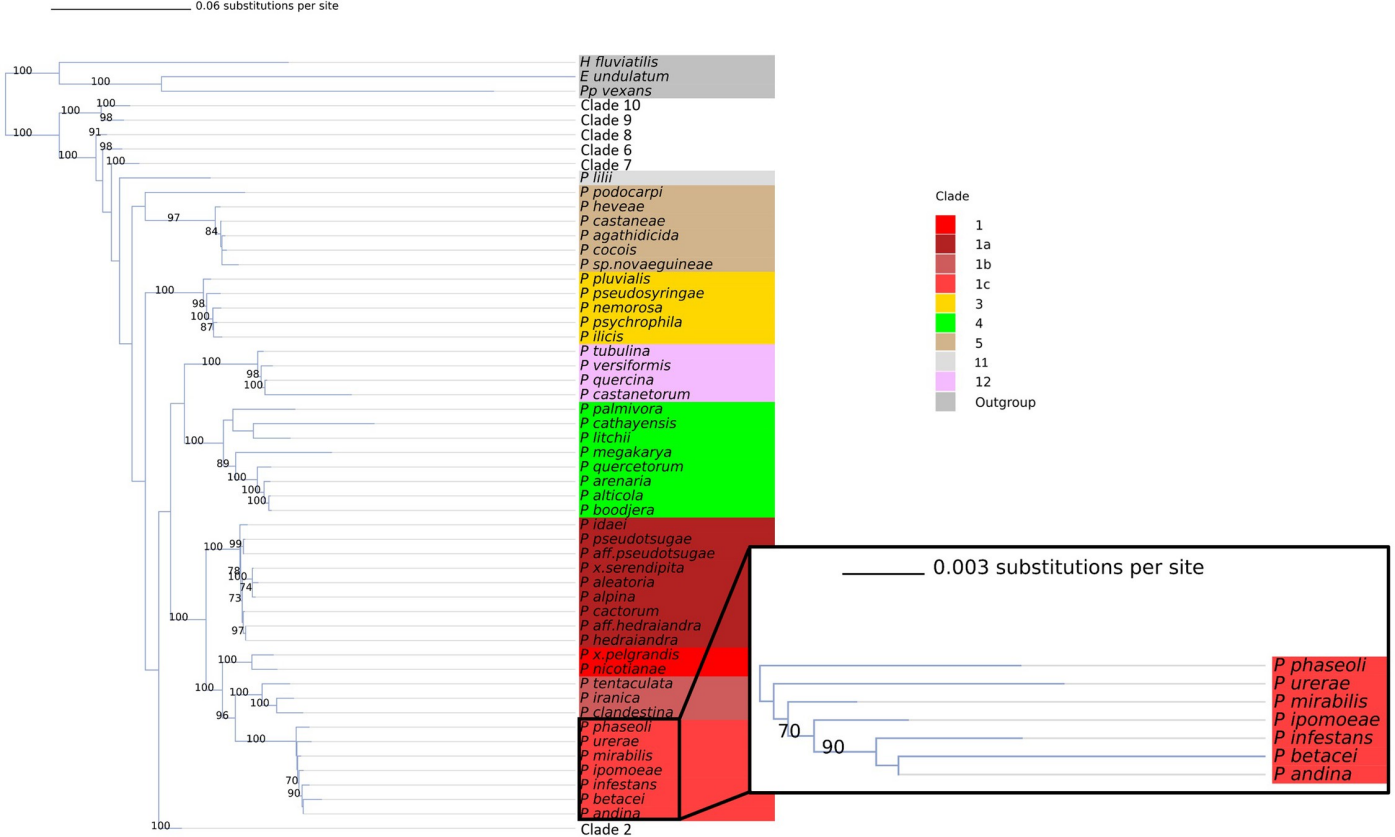
Phylogeny of clades 1, 3 4, 5, and 12. Collapsed phylogeny of the genus *Phytophthora* showing clades 1 (red), 3 (yellow), 4 (green), 5 (beige), and 12 (pink) in detail. Subclade values are shown as variations in color hue. Phylogeny is inferred using maximum likelihood and 1,000 bootstrap replicates for an alignment of 8 concatenated nuclear loci. Bootstrap values (in percent) are shown above each branch. The branches in the tree are drawn to scale and the scale bar represents 0.06 substitutions per site (0.003 substitutions per site in the inset).

Phylogeny-based placement of *P*. *acaciae* grouped it in clade 2d with sister species *P*. *bishii* (S5 Fig). *Phytophthora betacei* was placed in clade 1c near both *P*. *infestans* and *P*. *andina* (Fig 4).

Collaborator Inga Meadows tested the phylogenetic placement tool using 80 *Phytophthora* isolates spanning seven species (*P*. *nicotianae*, *P*. *tropicalis*, *P*. *palmivora*, *P*. *drechsleri*, *P*. *cryptogea*, *P*. *pseudocryptogea*, *and P*. *kelmania*) from other sequencing work. Phylogenetic placements matched previous identification, with the exception of eight isolates of *P*. *cryptogea* that were reclassified as *P*. *pseudocryptogea* using T BAS. In addition, species of *Phytophthora* reported on rhododendron were also correctly placed in the tree.

### Metadata

**Fig 5:**
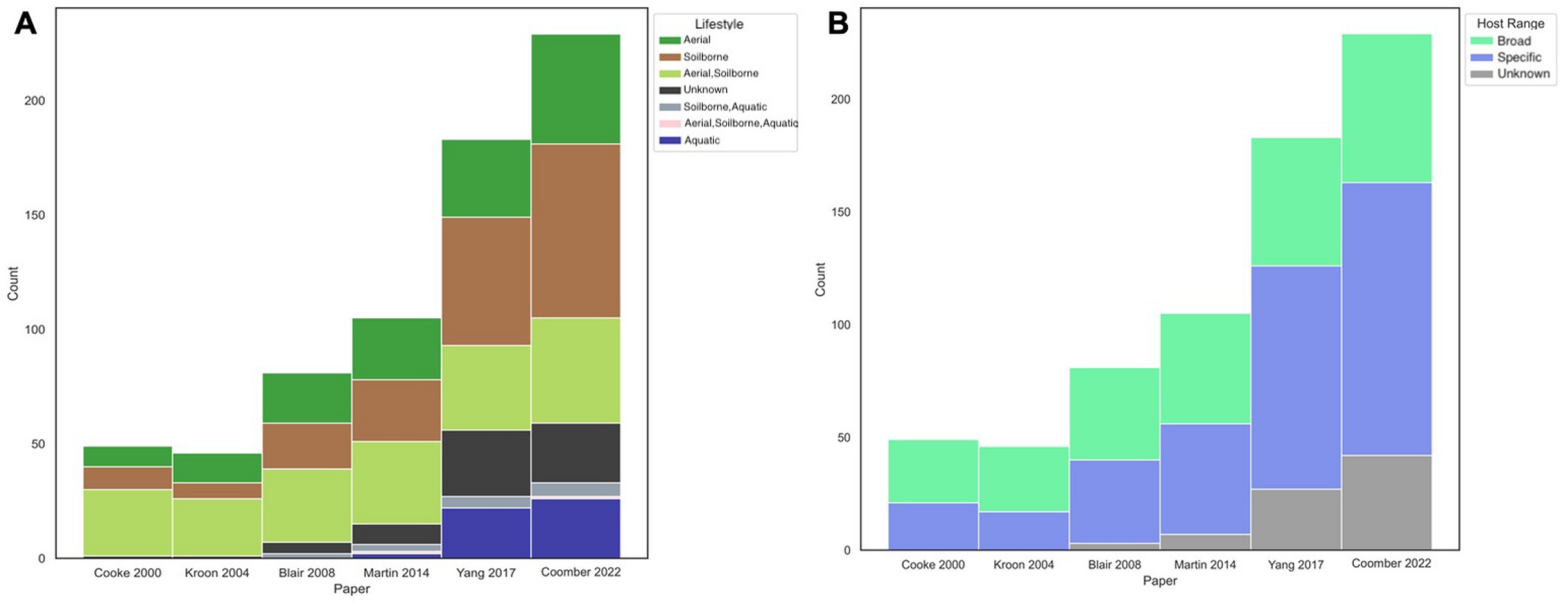
**(A)** Histogram of *Phytophthora* species in each lifestyle category over time. Histogram showing the number of *Phytophthora* species in each lifestyle category in major phylogenies published since 2000. **(B)** Histogram of *Phytophthora* species sorted by host ranges over time. Histogram showing the number of *Phytophthora* species in each host range category in major phylogenies published since 2000. Host specificity was characterized as “broad” or “specific”. Species with reported hosts in three or more plant families were characterized as "broad”, while species with reported hosts in two or fewer plant families were described as "specific”.

**Fig 6:**
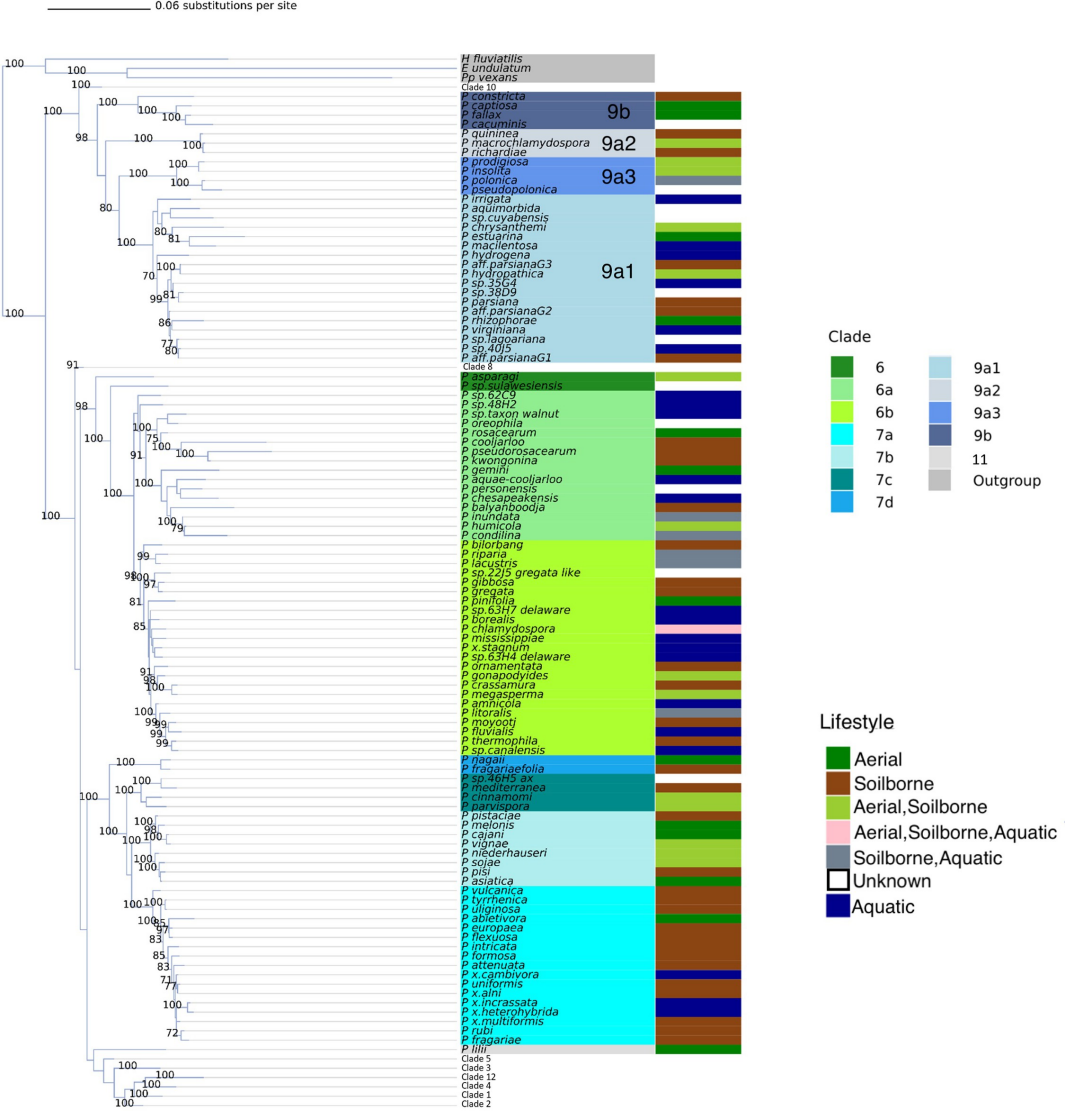
Phylogeny of *Phytophthora* clades 6, 7, and 9. Collapsed phylogeny of the genus *Phytophthora* showing 6 (green), 7 (teal), and 9 (blue) in detail. Subclade values are shown as variations in color hue. Phylogeny is inferred using maximum likelihood and 1,000 bootstrap replicates for an alignment of 8 concatenated nuclear loci. Bootstrap values (in percent) are shown for each branch. The branches in the tree are drawn to scale and the scale bar represents 0.06 substitutions per site.

Metadata, such as sexual reproductive strategies (heterothallic, homothallic, or sterile) were collated across the genus (S6 Fig). Since 2005 the number of homothallic species reported has exceeded the number of heterothallic species (S7 Fig). In general, the frequency of *Phytophthora* associated with different lifestyles (soilborne, aquatic, or aerial) has increased with additional species reported in each category over time (Fig 5A). Over the past 10 years, new *Phytophthora* species have been described more often from soilborne (includes root infecting species) than aerial lifestyles (Fig 5A). However, clusters of multiple aquatic species are present in Clades 6, 7, and 9 (Fig 6). In recent years, there has been a significant expansion in the number of surveys of water for *Phytophthora* species, likely explaining these results.

The level of host specificity also varies widely across the genus. Host specificity was characterized as “broad” or “specific” based on the number of reported hosts the pathogen has as well as the relatedness of the hosts. If a *Phytophthora* taxon was reported on three or more plant families it was characterized as “broad,” otherwise it was considered “specific.” It is important to note that reported hosts is not necessarily equivalent to host range as future pathogenicity trials could reveal additional hosts. This dichotomy in reported hosts of *Phytophthora* species was also acknowledged by Chen et al. (2021) in their recent phylogeny. Species with a broad list of reported hosts were clustered in Clades 1, 2, and 7 (Fig 2). However, the ability to cause disease on a wide variety of hosts is also generally widespread, being found at least once in all clades (Fig 2). In general, more recently described species fall in the “specific” reported host category than the “broad” reported host category, although this may change as these species are more thoroughly studied for host range (Fig 5B).

### *Phytophthora infestans* SSR classifier

In order to differentiate clonal lineages within *Phytophthora infestans* we estimated a threshold genetic distance to separate lineages. This threshold genetic distance was estimated as 0.099 by Poppr’s cutoff prediction function. A histogram of all pairwise comparisons of Bruvo’s genetic distance for the included lineages was bimodal, indicating a population with mixed forms of sexual and asexual reproduction, as is characteristic of *P*. *infestans* (S1 Fig). Pairs with a Bruvo’s genetic distance below the threshold occurred in the first peak of the distribution and indicated clonal relationships. In other words, the pair consisted of clones of the same lineage or genotype. Above the threshold was another peak in the distribution of Bruvo’s distances. Pairs falling in this range of the distribution represent lineages, which are distinctly different, likely as a result of sexual recombination. In other words, these are lineages that should not be considered the same genotype. This result visually confirmed the threshold genetic distance as estimated by Poppr’s cutoff prediction function. The SSR classifier was parameterized to use a cutoff threshold of a Bruvo’s distance of 0.099 in order to consider an unknown *P*. *infestans* isolate a match to a described lineage.

The reference dataset for classifying genotypes consisted of 2,176 isolates of *P*. *infestans* and their corresponding microsatellite genotypes obtained from over thirty years of collecting by Jean Beagle Ristaino. The overall accuracy of the classifier was approximately 0.98, with an unweighted Kappa statistic of 0.977. Various summary statistics were also calculated for each individual class (lineage, n = 36) that isolates could be placed into (S3 Table). Some of the isolates included in the reference dataset had one or only a few representative genotypes. For these isolates, accuracy, precision, and other summary statistics were not calculated because there were no closely matching isolates. Many of these isolates are no longer in circulation (most of the US lineages except US-23, US-8, and US-11) or were transient. Importantly, the SSR classifier was able to identify the US genotypes from the recent past that are currently circulating (S3 Table). As collection of isolates continues, the classifier will continue to grow more robust with the increase in reference data. A list of all representative genotypes used to develop the classifier and their SSR profiles can be found in S4 Table. This includes several genotypes for which there were too few isolates to perform statistical testing.

To evaluate the performance of the *P*. *infestans* classifier with incomplete SSR genotype data, a testing dataset was developed that included genotyped lineages which had at least 7 but no more than 11 SSR loci genotyped. Many of these isolates were from historic samples of the FAM-1 lineage. For incomplete data, overall accuracy was 0.968 and the Kappa statistic was 0.954.

Out of all the genotypes in the dataset, the SSR classifier was unable to distinguish between some genotypes at the decided threshold. Most of these multi-genotype groups contain one genotype that is still in circulation, as well as some genotypes that are no longer found. In this case, the genotype that is still in circulation is returned. If the multi-genotype group contains multiple genotypes still in circulation, all those genotypes are returned after querying the database. For example, genotypes US-6 and US-7 could not be distinguished from US-11 by our classifier. However, US-6 and US-7 were transient lineages that sexually recombined to produce US-11 [96, 97]. Of these three lineages, only US-11 has persisted and so this is the lineage name that is returned by the classifier.

## Discussion

### Genus tree placements

The TBAS *Phytophthora* tree presented here is designed to serve as a starting point for a curated, living phylogeny of the genus that may include species that are the direct ancestors of other contemporary taxa. Including at least three nuclear loci for well-described *Phytophthora* taxa and additional, newly described taxa has allowed for the resolution of conflicts within the genus phylogeny. Setting a minimum of three nuclear loci allowed for the inclusion of *Phytophthora* taxa that have been described that have few sequenced loci, while still maintaining the robustness of the tree. The loci included vary from species to species depending on what sequence data is available for each species, but all include some of the eight nuclear loci listed above. The nine loci used in this study all have utility in differentiating *Phytophthora* species, but the utility of each locus varies across the *Phytophthora* clades. [32]. Here, we found that including at least three nuclear loci (we recommend *Btub*, *TigA*, and *ITS*) allowed for the differentiation of all the species included in our tree. For species identification, we recommend using both a nuclear placement comprising at least three loci and placement into the mitochondrial *CoxI* tree. Hybrid *Phytophthora* taxa can be challenging to distinguish from their parental taxa, as is seen with the *P*. x *alni* species complex in our phylogenies [15, 48, 101].

This phylogeny is current at the time of submission of this publication. As new species are described researchers can query the tree and request placement of the species into the phylogeny by sending an email to us and following the submission guideline in the tutorial (S1 File).

Our work has resolved some issues of clade placement of some species and expanded the currently published phylogenies. For example, the placement of *P*. *quercina* has varied since its inclusion in the first molecular phylogeny of the *Phytophthora* genus [25]. Cooke et al. placed *P*. *quercina* into clade 3 with *P*. *ilicis* [25]. In their 2008 phylogeny, Blair and colleagues placed *P*. *quercina* in clade 4 with *P*. *arecae*, *P*. *megakarya*, *P*. *palmivora*, and *P*. sp. *“quercetorum”* [27]. In 2014, Martin and colleagues found *P*. *quercina* to be in its own clade with *P*. sp. *ohioensis* and not part of the canonical clade system [27]. In 2017 Yang and colleagues grouped *P*. *quercina* along with *P*. sp. *ohioensis* back into clade 4 [14]. On IDPhy, *P*. *quercina* is categorized as part of clade 3b in the ITS phylogeny [33]. Recently several new relatives of *P*. *quercina*, *P*. *versiformis*, *P*. *tubulina*, and *P*. *castanetorum*, have been described. By incorporating these new sister species and using a multilocus approach, it has been confirmed that these four species comprise a new clade 12 [13, 16, 63]. The phylogeny presented here supports this conclusion, with *P*. *quercina*, *P*. *versiformis*, *P*. *tubulina*, and *P*. *castanetorum* forming a unique monophyletic clade referred to as clade 12. This clade is separate and distinct from the rest of clade 3, forming a sister group to clades 1 and 4 (Fig 3).

In their 2017 *Phytophthora* phylogeny, Yang and colleagues subdivided clade 9 into clades 9a and 9b, where 9b is monophyletic and 9a consists of three monophyletic subclades, 9a1, 9a2, 9a3 [14]. The addition of new species in our work supports this subdivision of clade 9 (Fig 6). Several species newly included in this phylogeny were previously characterized as only “clade 9” but here, we find support for a specific subclade (Fig 6 and Table 1). *Phytophthora cacuminis* was strongly supported as part of Clade 9b [74]. *Phytophthora pseudopolonica*, which was previously classified as clade 9, grouped closely with *P*. *polonica* in clade 9a3 [90]. The new species *P*. *prodigiosa* also clustered in clade 9a3, despite being classified as clade 9b by previous ITS-based phylogenies [33, 85]. *Phytophthora estuarina*, *P*. *sp*. *lagoariana*, *P*. *sp*. *cuyabensis*, and *P*. *rhizophorae* were all supported as members of clade 9a1 [79]. In general, the discovery of new species in clade 9 supports the current grouping of clade 9 into the subclades proposed by Yang et al. 2017 and by Chen et al. 2021 (Fig 6).

Clade placement was also confirmed for two species that we sequenced for this study including *P*. *acaciae* and *P*. *betacei*. *Phytophthora acaciae* was placed in clade 2d with close relatives *P*. *bishii* and *P*. *frigida*, which is in agreement with our previous work on this species (S5 Fig) [45]. *Phytophthora betacei* was confirmed as a member of clade 1c as previously reported (Fig 4) [46].

The mitochondrial *CoxI* locus was included in part because it has been a popular gene to use in barcoding in previous *Phytophthora* phylogenies [29, 30]. The topology of the *CoxI* mitochondrial tree differed from the nuclear derived trees, potentially because of uniparental mitochondrial inheritance, horizontal gene transfer, incomplete lineage sorting, and phylogenetic artifacts (S3 and S4 Figs). The mitochondrial tree we produced uses only the *Cox*1 locus, which provides less opportunity to differentiate haplotypes than a more detailed multilocus analysis. However, the C*ox*1 locus evolves much faster than some of the nuclear loci we used, so when used in combination with the nuclear genes, this locus may be useful to differentiate new haplotypes or new species. The *CoxI* locus was included as a single locus tree in theT BAS tool and is also available for use. It is not concatenated with the other nuclear loci in the multilocus tree.

The CoxI inferred tree is available for further analysis (S3 Fig and S1 Table). The presence of unexpectedly close relationships between *CoxI* loci for some species warrants further investigation into known and potential hybrids. For example, in the tree based on eight nuclear loci *P*. x *serendipita* is most closely related to *P*. *aleatoria* and *P*. *alpina* with very strong support (bootstrap value of 100) (Fig 4). However, in the tree inferred from the *CoxI* mitochondrial locus, *P*. x *serendipita* is a sister group to *P*. *hedraiandra* with very strong support (bootstrap value of 95) (S3 Fig). This indicates that *P*. x *serendipita*’s mitochondrial genome is more closely related to that of *P*. *hedraiandra* than the nuclear genome [95]. *P*. x *serendipita* is thought to be a hybrid of *P*. *hedraiandra* and *P*. *cactorum* [95]. Inheritance of the mitochondria from the *P*. *hedraiandra* parent would explain why it is closer to this species in the mitochondrial-based tree [95]. This is the case for the isolate included in our trees, but other isolates of this species may have *P*. *cactorum* as their mitochondrial parent and therefore place with *P*. *cactorum* in the mitochondrial phylogeny. More data, especially sequence data from additional mitochondrial loci, need to be collected for some species to further investigate potential hybridizations. Full mitochondrial genome genealogies would also be useful. The discordance of the *CoxI* tree with the other single locus nuclear trees, as well as previously published trees, led us to exclude the *CoxI* locus from our final tree (S4 Fig). Conflict between multilocus analysis and *CoxI* placement was also observed by Yang and Hong in their 2018 evaluation of *Phytophthora* markers [32].

Recently, it has also been proposed that the *rps10* mitochondrial locus be used to barcode *Phytophthora* species [98]. Although both *CoxI and rps10* can differentiate many *Phytophthora* species, it is important to include nuclear barcodes as well [63]. The frequency of hybridization events in the genus *Phytophthora* merits careful examination of both nuclear and mitochondrial genealogies in order to understand phylogenetic relationships [24, 94, 99, 101, 102, 103]. The addition of more mitochondrial loci into the *CoxI* tree such as *rps10* might strengthen support for these relationships, which could further clarify potential hybridization events. The phylogenomic computational tools developed by Van Pouke et al, will also be useful for understanding species hybrids in the genus [24]. We recommend separate placements of genes of interest on both the nuclear and mitochondrial trees and examination of relationships on each.

There is a lack of strong support in the both the nuclear and *CoxI* tree for the relationships between some species in the 1c clade. In the nuclear tree, there is strong support (bootstrap value of 90) for *P*. *infestans*, *P*. *betacei*, *and P*. *andina* forming a clade. However, the relationship between the next most closely related species, *P*. *mirabilis*, and these species, has weak support (bootstrap value of 70). Although the *CoxI* tree can also be used to inform these relationships, the relationships are not resolved with strong support in this tree either, indicating the need for more mitochondrial genome sequences. Given the recent addition of two new species in clade 1c, *P*. *urerae* and *P*. *betacei*, more whole genome sequence data and whole mitogenome data [98] will be needed to confidently understand the evolutionary relationships among 1c clade species. This clade is economically relevant and important in evolution studies and studies on the origins of the *P*. *infestans*. The inability to separate *P*. *betacei* from *P*. *andina* with strong support in our tree indicates that these species may be conspecific. A more comprehensive, genomic phylogeny of species in this clade should be conducted to strengthen confidence in the phylogeny. A finer resolution of the evolutionary relationships of *P*. *andina* to *P*. *betacei* is in progress in a related genome sequencing project in our lab.

Recent evidence has also shown that many genera of downy mildews place phylogenetically within the *Phytophthora* genus [63]. The downy mildew species are not included in the resource presented here because they are outside the scope of this project. We also did not expand to the provision 16 clade system. Although the genus *Phytophthora* is paraphyletic in that it includes the downy mildews in several nested clades within the genus tree [63], it has been proposed that the genus name *Phytophthora* be retained because it is both biologically and structurally cohesive [15]. We agree with this work. Evidence for the cohesion of the *Phytophthora* genus and the lack of distinguishing morphological or lifestyle traits to separate out the individual species within *Phytophthora* clades is furthered by our work. Many distinguishing traits, such as reproductive mode and ecological “life style”, appear to be spread across clades in the genus without a strong pattern. This indicates a shared evolutionary relationship between all of the species in the *Phytophthora* genus, regardless of later convergent evolution into multiple groups of downy mildews.

### Metadata

Incorporating the metadata into the phylogenetic visualization allows the user to see relationships in biological characteristics across the phylogeny. For example, the recent proliferation in the description of species that were isolated from aquatic habitats is shown to have occurred in multiple clades (Fig 2). The primary lifestyle of *Phytophthora* species (broadly aerial, soilborne, or aquatic) are widely distributed, with small clusters of taxa that have different lifestyles appearing in multiple clades (Fig 2). Several recently described species from aquatic systems are clustered together in clade 6a and clade 9 (Fig 6). It appears that the ability to have success in different substrates has evolved multiple times in the genus.

Other than clade 5 and clade 3, which have entirely homothallic species, every clade has representatives of both homothallic and heterothallic species (S6 Fig). Both homothallism (self-fertile) and heterothallism (outcrossing) have been hypothesized to be the basal state of the genus [25, 28]. Blair et al. 2008 agreed with Kroon and colleague’s assessment that homothallism was the basal state based on the lack of heterothallism in both Clades 9 and 10, the basal clades [27]. Here we show that heterothallic species are also present in these clades including *P*. *intercalaris* in clade 10 and *P*. *irrigata* and others in clade 9 (S6 Fig). The ancestral reproductive mode in *Phytophthora* is therefore unclear as more than 12 transitions from one state to another are shown in our most supported tree (S6 Fig). Thus, reproductive strategy may be more transient than previously assumed. Others have shown that environmental stimuli including stress, aging and even fungicides can shift species such as *P*. *infestans* from heterothallism to homothallism [100,101].

Reported host range from broad to specific is also a variable trait in *Phytophthora* and has been shown here and in other work [15] (Fig 2). Again, this indicates relative ease in the evolutionary switch from wide to narrow host range strategy. Additionally, *Phytophthora* species have been clustered into two invasiveness groups based on host range as either generalists or specialists [8]. The categorization of each taxon into broad or specific host range could provide useful information to determine the potential invasiveness of a given species and be of use to regulatory agencies attempting to conduct risk analysis [8, 10].

### Comparison to other databases

Several efforts have been made over the years to collect disparate information on *Phytophthora* species across the genus to facilitate identification, phylogenetics and evolutionary research. Notable examples include IDPhy, Phytophthora-ID for species and lineage identification, PhytophthoraDB and a lucid, dichotomous key for species identification [19, 23, 33–35].

IDPhy presents a wealth of morphological information and images and other metadata for species identification and is a valuable resource [16, 33]. This website contains fact sheets for *Phytophthora* species, morphological traits and molecular identification protocols to facilitate species identification. Sequence data for two genetic loci (*ITS* and *CoxI*) are also provided for many *Phytophthora* species as barcodes for species identification and can be blasted against via Genbank. In order to maintain compatibility with IDPhy, we have used the same sequence data for the *ITS and CoxI* regions for the 162 species identified as types or ex types in the IDPhy molecular key. We have also made an effort to synchronize metadata, such as lifestyle (soilborne, aerial, or aquatic) and distribution, with that provided by IDPhy.

Phytophthora-ID enables phylogenetic placement of unknown strains based on only 2 genes—*CoxI* and *ITS* sequences, and is not as robust as our tool [34]. However, Phytophthora-ID does not contain the full global set of known SSR lineages for *P*. *infestans* and many SSR lineages from the EuroBlight database are missing. The synthesis of genetic information with morphological characteristics and other descriptions is also not addressed by Phytophthora-ID.

Recently, the genus *Phytophthora* was included in the fourth edition of the “Genera of phytopathogenic fungi” series (GOPHY4) [31]. This included a whole genus tree of 192 species. However, this tree used a limited number of loci (up to four) from both the nuclear and mitochondrial genomes. *Phytophthora* species are known to undergo interspecies hybridizations, and including loci with discordant inheritance patterns can result in misleading phylogenies [102–104]. By including both nuclear and mitochondrial loci, but in separate phylogenies, we can mitigate these issues.

With a rapidly expanding genus, the need to update phylogenies and metadata descriptions to include newly described species and provisional species is critical. This tool will be useful to post provisional new species and give researchers access to expand phylogenetically new or interesting understudied clades of *Phytophthora* as a community. The contribution of the database presented here is to synthesize the wealth of sequence data and information that has been collected on *Phytophthora*, and to provide a more robust phylogenetic framework for the genus that can be updated at the pace of discovery by researchers in the community. In contrast to other resources which use a single gene e-barcoding strategy for quick identification, we have developed a more detailed phylogenetic tool.

### Future directions

By incorporating the *Phytophthora* genus phylogeny into the T-BAS system, researchers will be able to add metadata, sequence data, and taxa as new species are described and our understanding of the genus continues to expand. Metadata that is not presented here, such as taxonomic characteristics or phenotype data, could easily be added to the tree if of interest to the larger community. Adding a new species will require submission of the metadata and sequence data through the T-BAS website so the data can be curated before uploading. Maintaining a robust and rapidly updating phylogeny will allow the streamlining of scientific information on the genus to keep pace with the discoveries of new species and characteristics. Incorporating the metadata directly into the phylogeny may also provide new insights for *Phytophthora* researchers of where new outbreaks are occurring. We plan to expand the mapping capabilities within the T-Bas tool. Thus, a community of research experts involved in Phytophthora research will need to be designated to become validators for the genus level phylogeny and the *P*. *infestans* classifier.

Although the *Phytophthora* genus tree enables rapid phylogenetic placement of newly sequenced isolates, phylogenetic placement alone cannot be used for species level identification. In addition to sequencing the loci used in this study for phylogenetic placement in T-BAS, other methods such as morphology should be used to verify the species an isolate belongs to. Moving forward, many newly described species need to be more thoroughly studied. Several of the newly described species in this phylogeny have only a few sequenced loci, which weakens phylogenetic placement. For example, clade 9 remains difficult to fully resolve in part because only 3–4 of the loci are sequenced for several new species (*P*. *psuedopolonica*, *P*. *cacuminis*, *P*. *estuarina*, and *P*. *rhizophorae*). Furthermore, key metadata characteristics for many species, such as sexual strategy and hosts are missing. Going forward, we recommend at least three of the nuclear loci utilized here be sequenced for new species descriptions in addition to morphological requirements. Placement in both the nuclear and mitochondrial trees is also recommended for species identification. In choosing which loci to include at a minimum, we suggest consulting Yang and Hong (2018) to select what loci might be most useful for the clade(s) of interest. Significant differences at these loci and in morphological traits is needed to warrant the description of a new species. These requirements will clarify the existing phylogeny and enable more robust phylogenetic placements of new species. Adding information into the phylogeny is facilitated by the T-BAS system, so new information about *Phytophthora* species can be readily available to the community, without the need to publish an updated description.

A review of the phylogeny also shows that newer species are often being described from natural ecosystems, such as riparian buffers, aquatic and forested areas, as opposed to agricultural systems. Further exploration of these systems is warranted, as it will likely reveal more species of *Phytophthora* that are yet to be discovered [8].

The utility of this tool for the plant-pathogenic *Phytophthora* genus serves as a proof of concept for other pathogen phylogenies. A separate live phylogeny for downy mildew fungi would be a great additional tool for the oomycete research community. Enhancing pathogen phylogenies with metadata and live taxon placement will facilitate research into a diversity of pathogen species. This tool also emphasizes the sharing and standardization of data–including the phylogeny, multiple sequence alignments and sequence data, biological sample data, specimen vouchers and other associated metadata. The comprehensive nature of this tool has been enabled by the wealth of published *Phytophthora* research that has been collected and shared by our research group and many other groups. We intend to contribute to and facilitate this trend by synthesizing these data streams. The efficacy of the live phylogeny presented here depends on open data sharing of not only the database tool but of cultures of newly described species between researchers and deposit in respected culture collections by the global *Phytophthora* research community. Many but not all of the species described herein are available to researchers from the Ristaino Phytophthora Collection at NC State.

## Conclusion

In conclusion, our work provides a curated, community-updated phylogeny for the genus *Phytophthora* incorporating both sequence data and biological metadata. This phylogeny will serve as a resource to the community as research on *Phytophthora* continues. Newly described species have been added to the phylogeny as a proof-of-concept, placing congruously with their species descriptions (Table 1). A microsatellite-based classifier for *P*. *infestans* genotypes was also developed to identify genotypes of *P*. *infestans* more rapidly. As the number of genotypes continues to grow and change, the research community too can update this tool to keep pace with discovery. This live phylogeny format can also be extended to a diversity of pathogens to facilitate phylogenetic placement of other pathogenic microbes.

## Supporting information

S1 Fig Histogram of Bruvo’s genetic distance between *P*. *infestans* isolates. (DOCX)

S2 Fig Histogram of *Phytophthora* species per clade. (DOCX)

S3 Fig *CoxI* based *Phytophthora* phylogeny. (DOCX)

S4 Fig Mesquite Hypha comparison of single locus phylogenies. (DOCX)

S5 Fig Detailed phylogeny of *Phytophthora* clade 2. (DOCX)

S6 Fig *Phytophthora* phylogeny with sexual characteristics highlighted. (DOCX)

S7 Fig Histogram of *Phytophthora* species per sexual characteristic. (DOCX)

S1 Table Accession numbers for sequence data used in this study. (XLSX)

S2 Table PCR Primers and detailed protocol. (DOCX)

S3 Table *P*. *infestans* classifier statistics. (DOCX)

S4 Table Lineages in *P*. *infestans* classifier. (XLSX)

S1 File T-BAS tree placement tutorial. (DOCX)

S2 File *P*. *infestans* classifier tutorial. (DOCX)
