# Supplementary material for: An open-access T-BAS phylogeny for emerging Phytophthora species
Source: PLoS One. 2023 Apr 3;18(4):e0283540. doi: 10.1371/journal.pone.0283540 (PMC10069789; doi:10.1371/journal.pone.0283540)
Supplement: S1 Fig — (DOCX) [file pone.0283540.s001.docx]

S1 Fig. Histogram of Bruvo’s genetic distance of all pairwise comparisons of isolates of *Phytophthora infestans* included in the SSR classifier reference dataset. The bimodal distribution is typical of species with a mixed reproduction systems like *Phytophthora infestans*. The first peak represents pairs which are closely related and derived from clonal reproduction. The second peak consists of pairs which are related via sexual recombination.


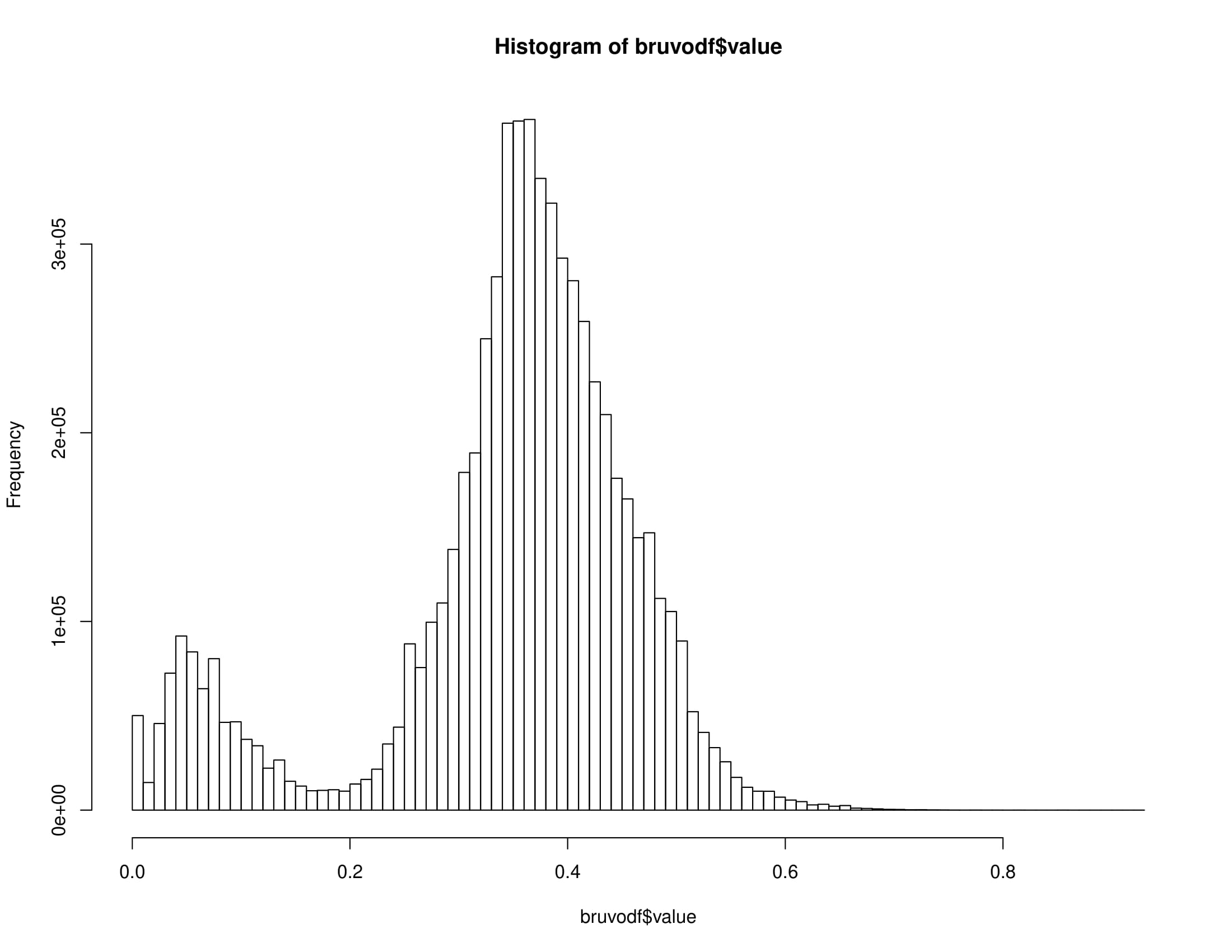


Genetic distance
