# Supplementary material for: An open-access T-BAS phylogeny for emerging Phytophthora species
Source: PLoS One. 2023 Apr 3;18(4):e0283540. doi: 10.1371/journal.pone.0283540 (PMC10069789; doi:10.1371/journal.pone.0283540)
Supplement: S2 Fig — (DOCX) [file pone.0283540.s002.docx]

S2 Fig. Histogram showing the number of species in each clade in major *Phytophthora* phylogenies published since 2000. Subclade distinctions have been removed for simplification.


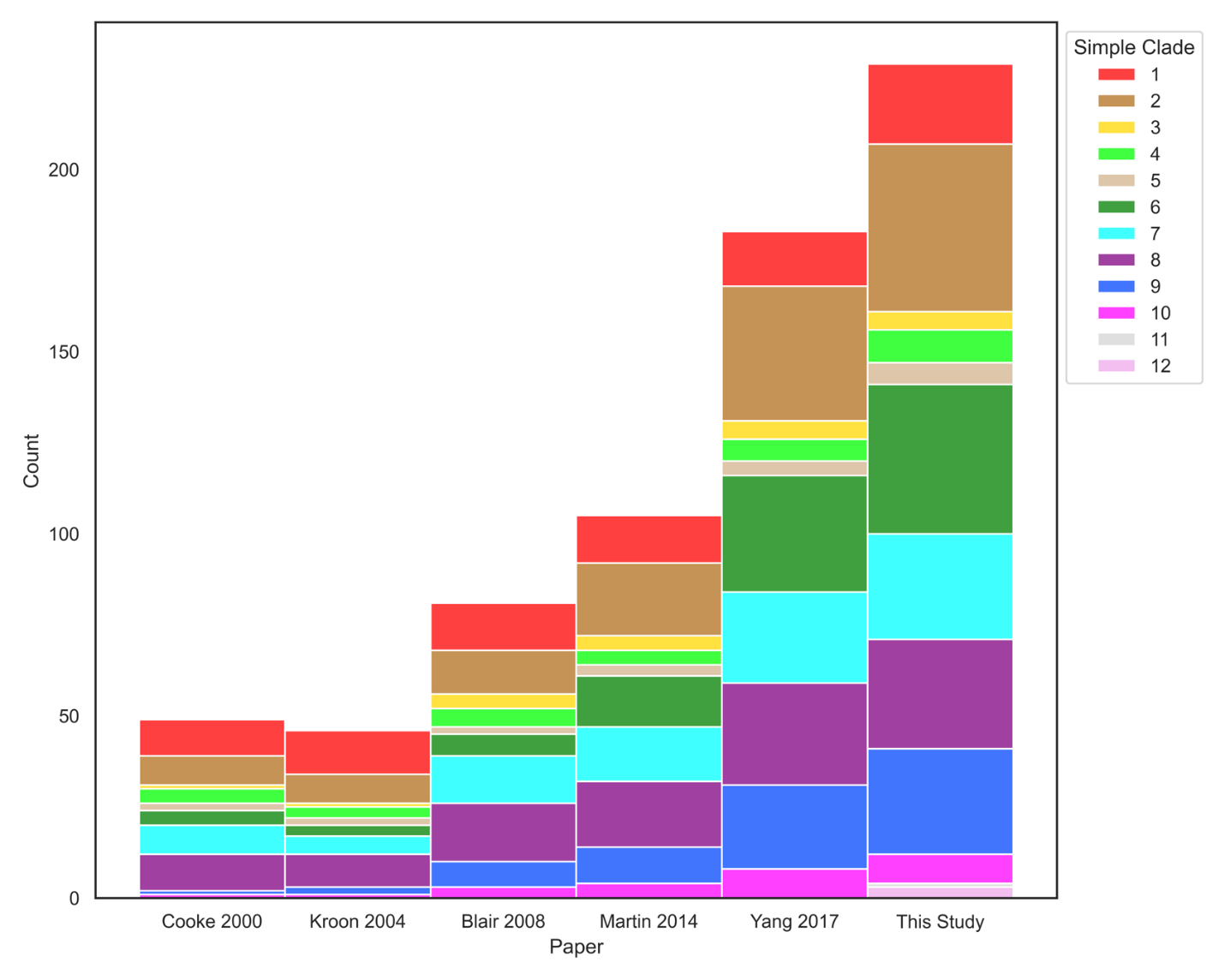


Coomber 2023
