# Supplementary material for: An open-access T-BAS phylogeny for emerging Phytophthora species
Source: PLoS One. 2023 Apr 3;18(4):e0283540. doi: 10.1371/journal.pone.0283540 (PMC10069789; doi:10.1371/journal.pone.0283540)

S3 Fig. Phylogeny of the genus *Phytophthora* inferred using maximum likelihood and 1,000 bootstrap replicates for the mitochondrial *CoxI* locus. Clade and subclade values are shown as variations in color hue. Bootstrap values (in percent) are shown for each branch. Branch lengths are drawn proportional to number of substitutions.


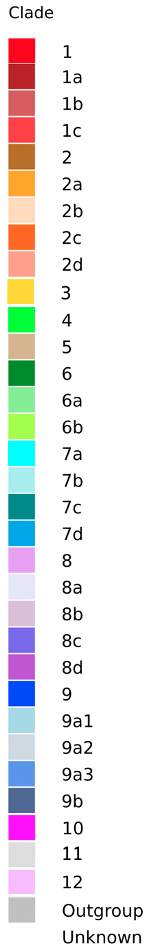

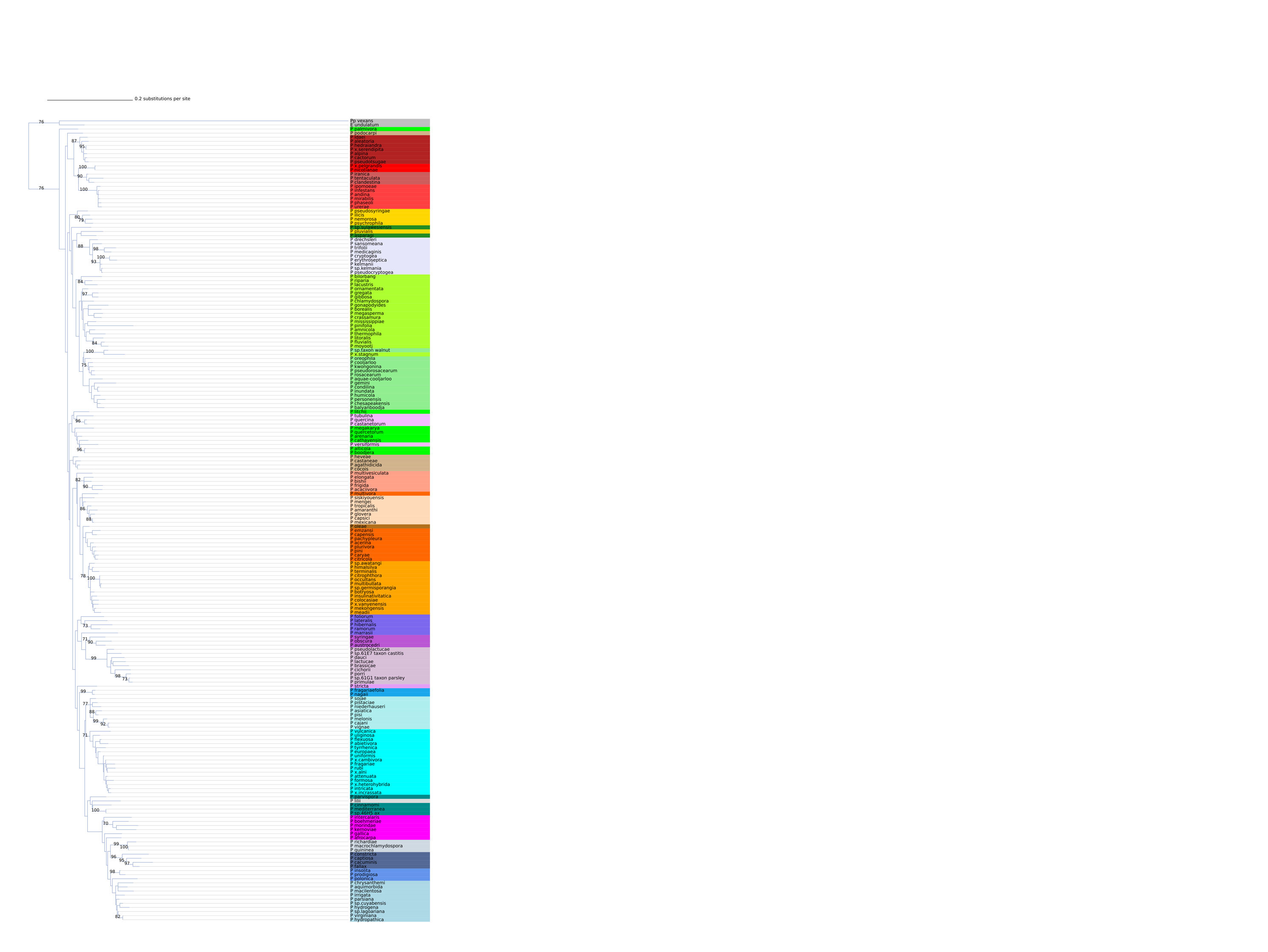

Supplement: S3 Fig — (DOCX) [file pone.0283540.s003.docx]
