# Supplementary material for: An open-access T-BAS phylogeny for emerging Phytophthora species
Source: PLoS One. 2023 Apr 3;18(4):e0283540. doi: 10.1371/journal.pone.0283540 (PMC10069789; doi:10.1371/journal.pone.0283540)

S4 Fig. Comparison of nine single-locus maximum likelihood phylogenetic trees for the *Phytophthora* genus inferred using Mesquite Hypha. A phylogenetic tree inferred using all eight nuclear loci (i.e., total evidence tree) serves as the backbone tree for comparison. Concordance or discordance between the trees for each locus is shown in grids at each node. Position within the grid corresponds to locus, and box color corresponds to the level of conflict or agreement. Bootstrap values for the total evidence tree are shown for each branch. Branch lengths are drawn proportional to number of substitutions.


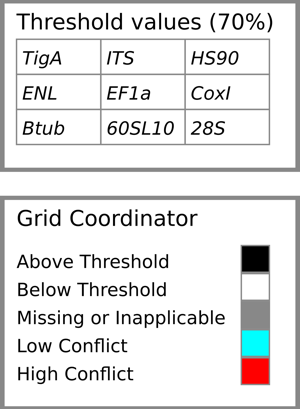

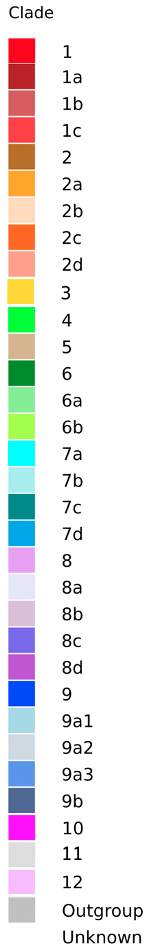

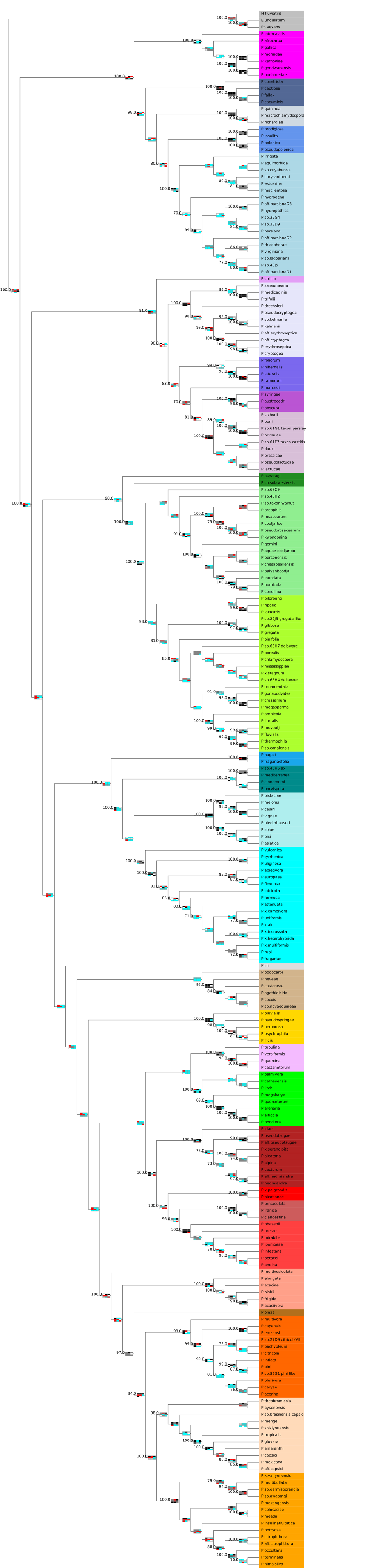


S4 Fig Continued


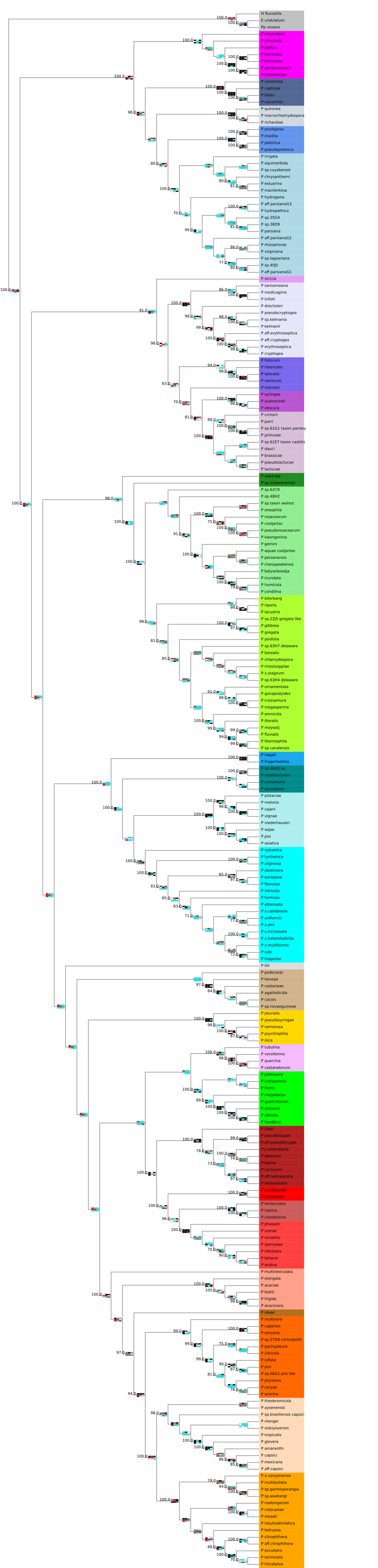

Supplement: S4 Fig — (DOCX) [file pone.0283540.s004.docx]
