# Supplementary material for: An open-access T-BAS phylogeny for emerging Phytophthora species
Source: PLoS One. 2023 Apr 3;18(4):e0283540. doi: 10.1371/journal.pone.0283540 (PMC10069789; doi:10.1371/journal.pone.0283540)

S5 Fig. Collapsed phylogeny of the genus *Phytophthora* showing clade 2 (orange) in detail. Subclade values are shown as variations in color hue. Bootstrap values are shown for each branch. Branch lengths are drawn proportional to number of substitutions. Phylogeny is inferred using maximum likelihood (RaxML) for 8 concatenated nuclear loci.


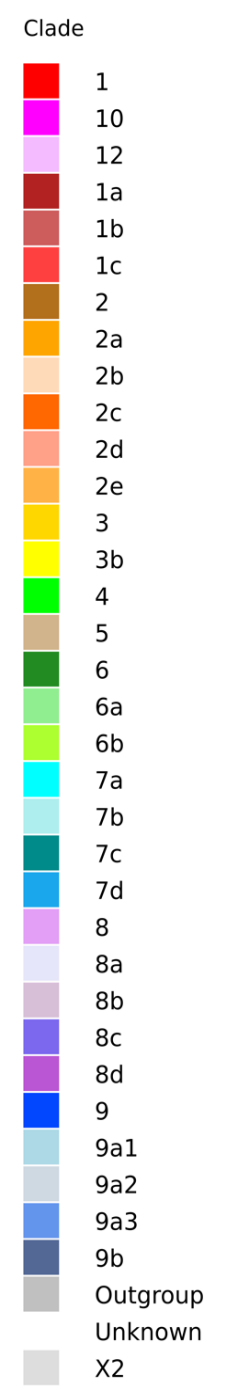

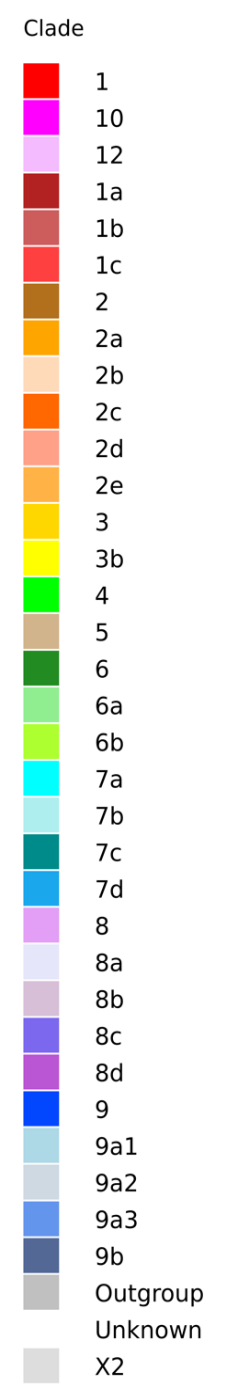

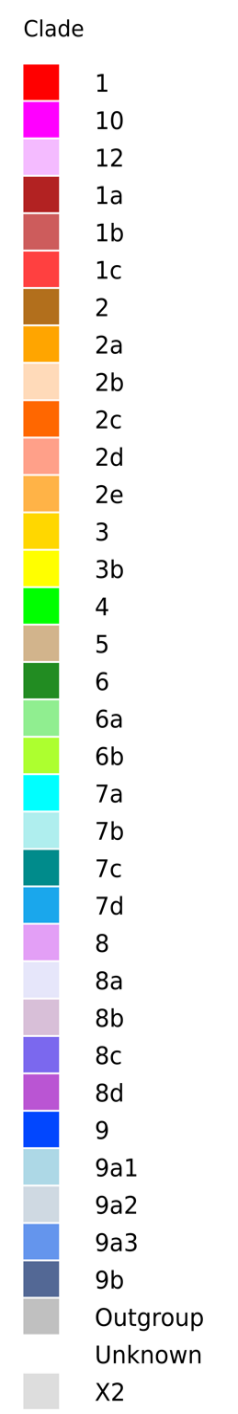


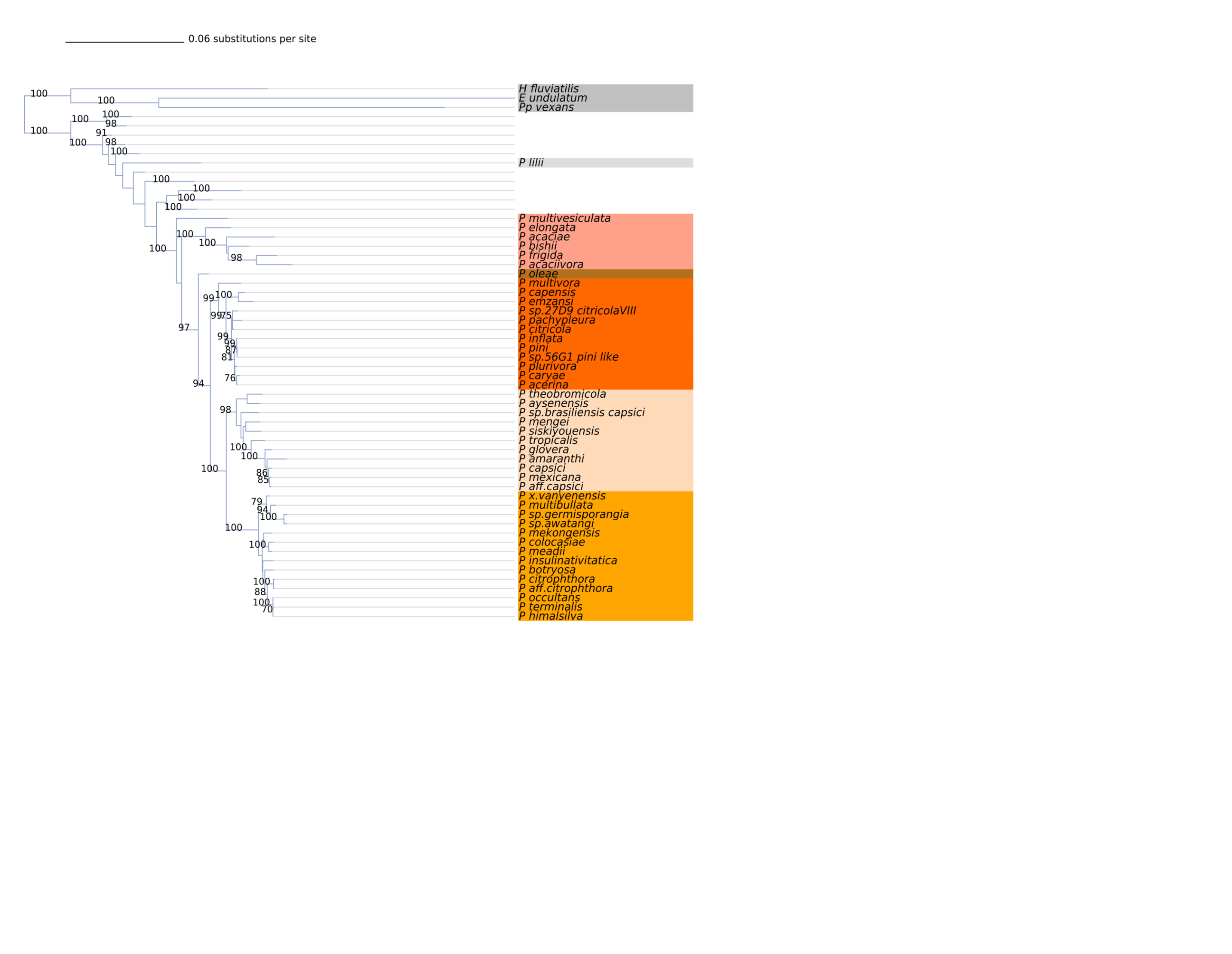

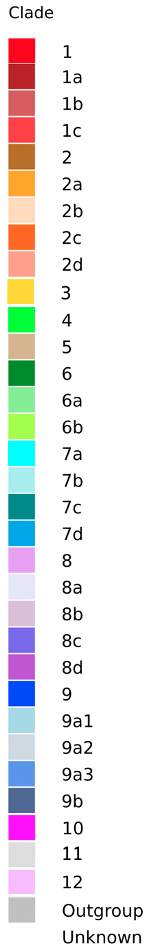

Supplement: S5 Fig — (DOCX) [file pone.0283540.s005.docx]
