# Supplementary material for: An open-access T-BAS phylogeny for emerging Phytophthora species
Source: PLoS One. 2023 Apr 3;18(4):e0283540. doi: 10.1371/journal.pone.0283540 (PMC10069789; doi:10.1371/journal.pone.0283540)
Supplement: S6 Fig — (DOCX) [file pone.0283540.s006.docx]

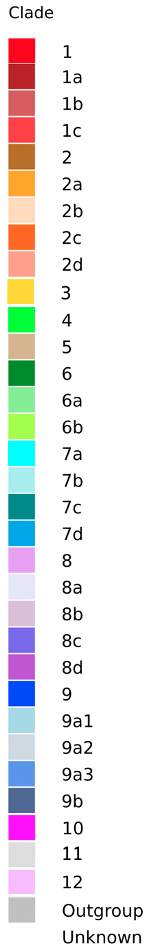
S6 Fig. Radial phylogeny of the genus *Phytophthora* inferred with maximum likelihood (RaxML) for an alignment of 8 concatenated nuclear genes. Coloring on the inner ring indicates clade. Coloring of the branches indicates reproductive mode (homothallic, heterothallic, or sterile). Branch lengths are not to scale.


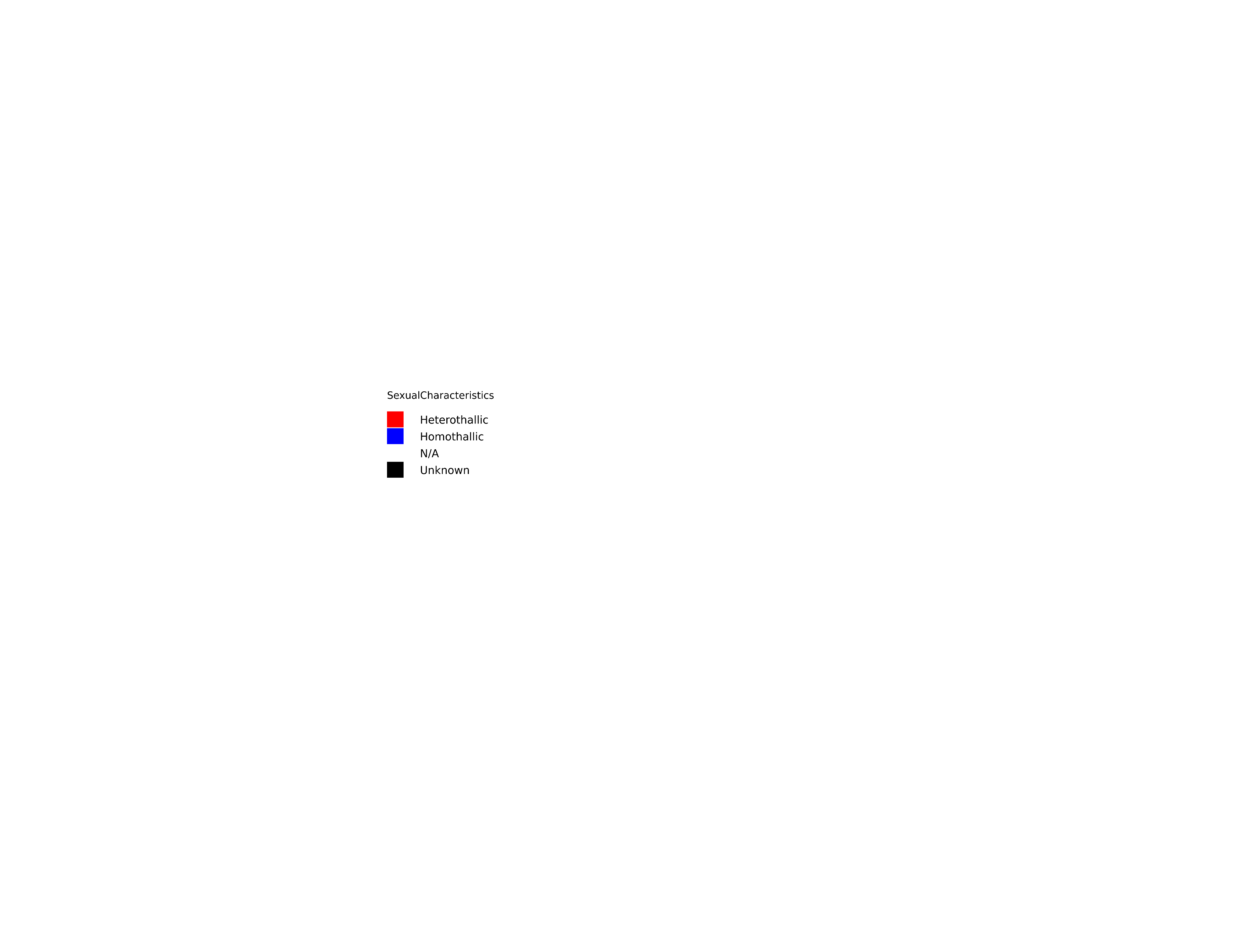

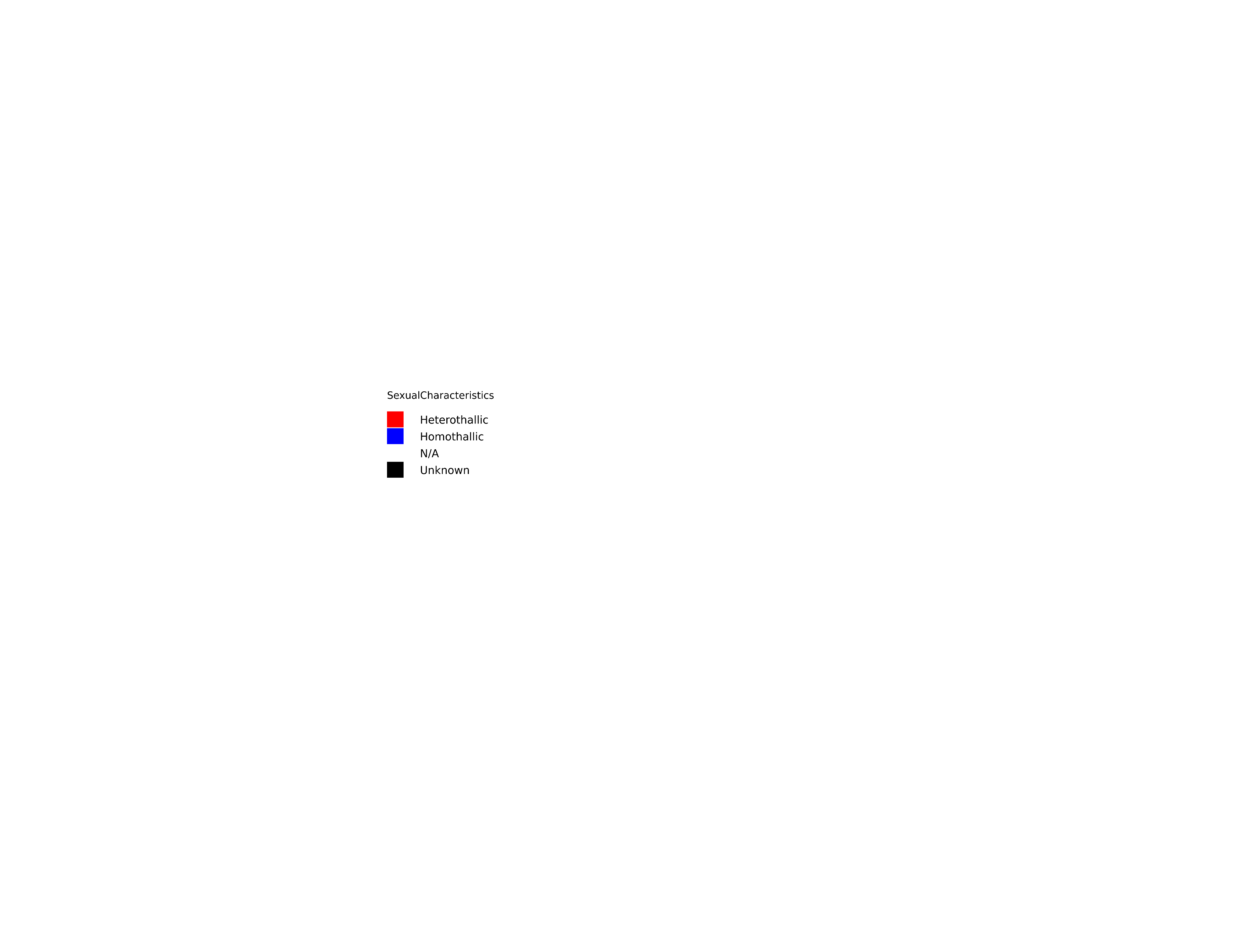

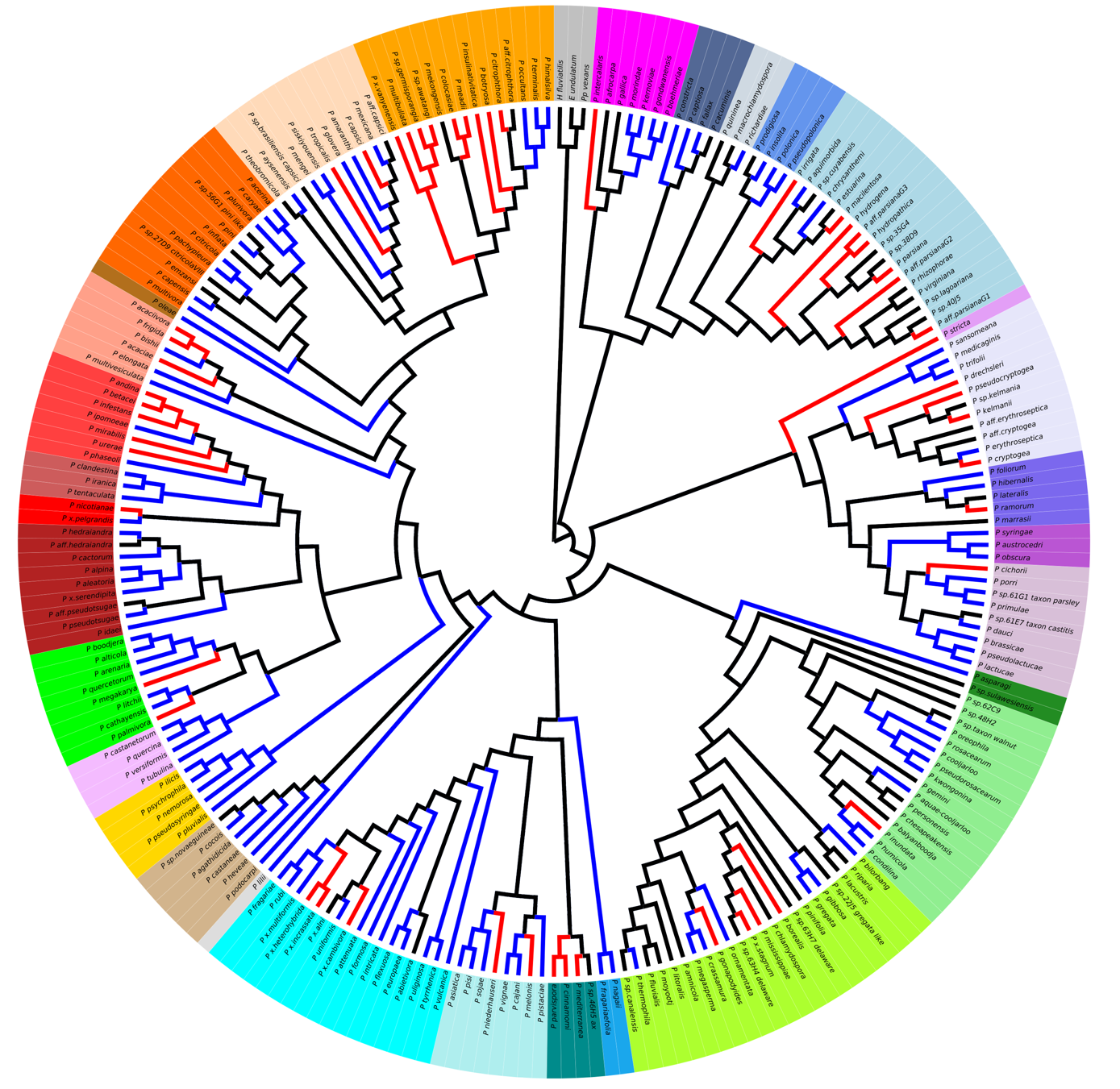


Reproductive Mode

Heterothallic

Homothallic

Sterile
