# Supplementary material for: An open-access T-BAS phylogeny for emerging Phytophthora species
Source: PLoS One. 2023 Apr 3;18(4):e0283540. doi: 10.1371/journal.pone.0283540 (PMC10069789; doi:10.1371/journal.pone.0283540)
Supplement: S7 Fig — (DOCX) [file pone.0283540.s007.docx]

S7 Fig. Histogram showing the number of *Phytophthora* species in each reproductive mode category in all major phylogenies published since 2000.


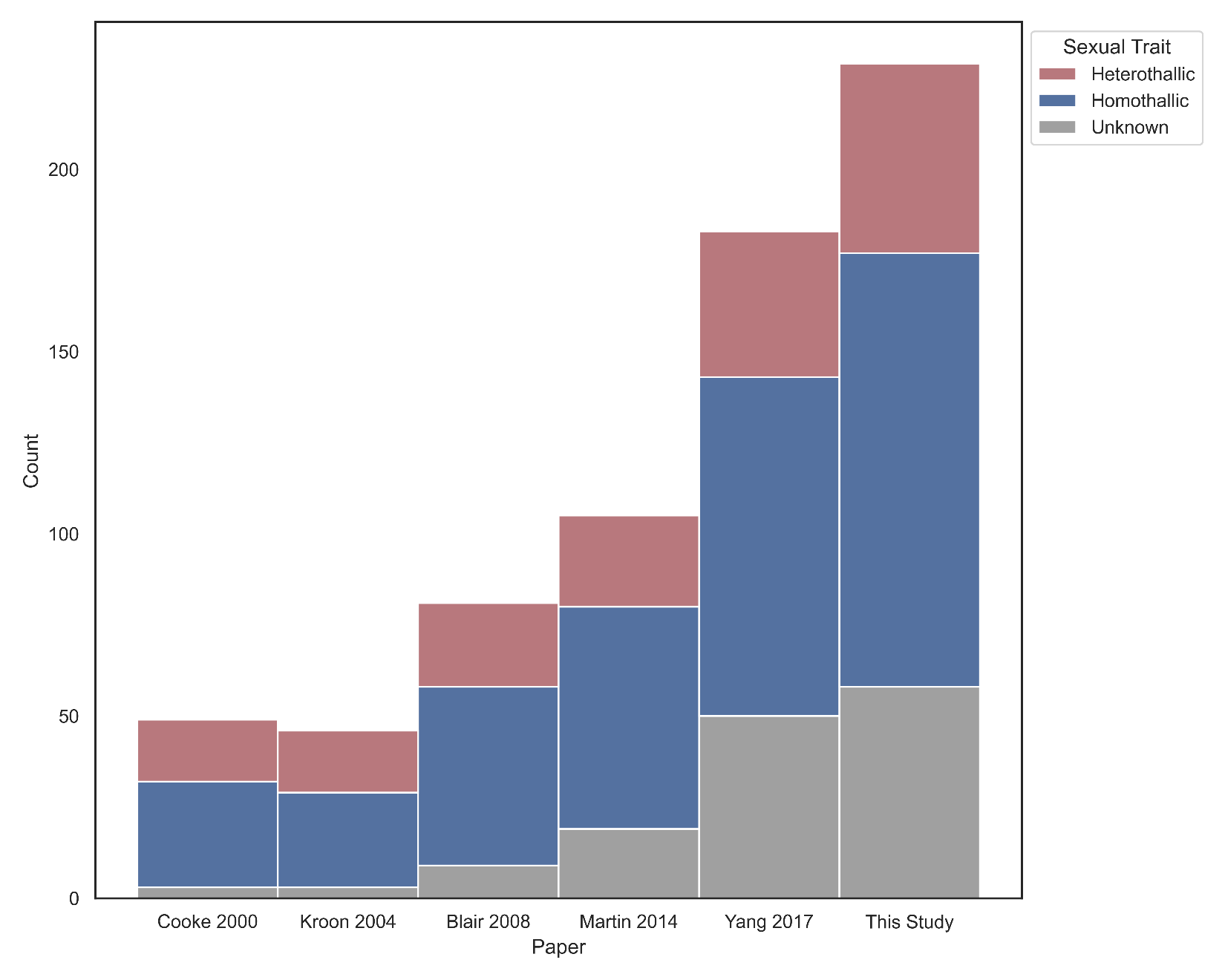


Coomber 2023
