# Supplementary material for: An open-access T-BAS phylogeny for emerging Phytophthora species
Source: PLoS One. 2023 Apr 3;18(4):e0283540. doi: 10.1371/journal.pone.0283540 (PMC10069789; doi:10.1371/journal.pone.0283540)
Supplement: S2 Table — (DOCX) [file pone.0283540.s009.docx]

**S2 Table**. Locus, primer name, sequences annealing temperature and source of primers used to amplify the nine loci used in this study.

| Locus | Forward Primer Name | Forward Primer Sequence | Reverse Primer Name | Reverse Primer Sequence | Annealing Temperature | Source |
| --- | --- | --- | --- | --- | --- | --- |
| *28S* | LROR-O | 5’- ACCCGCTGAACTYAAGC-3’ | LR6-O | 5’-CGCCAGACGAGCTTACC-3’ | 53ºC | [1, 2] |
| *60SL10* | 60SL10_for | 5'-GCTAAGTGTTACCGTTTCCAG-3’ | 60SL10_rev | 5’- ACTTCTTGGAGCCCAGCAC-3’ | 53ºC | [3] |
| *Btub* | Btub_F1 | 5’-GCCAAGTTCTGGGAGGTCATC-3’ | Btub_R1 | 5’- CCTGGTACTGCTGGTACTCAG-3’ | 60ºC | [3, 4] |
| *CoxI* | COXF4N | 5′-GTATTTCTTCTTTATTAGGTGC-3′ | COXR4N | 5′-CGTGAACTAATGTTACATATAC-3′ | 52ºC | [4] |
| *EF1a* | EF1A_FL | 5’- GGTCACCTGATCTACAAGTGC-3’ | EF1A_RL | 5’- CCTTCTTGTTCACCGACTTG-3’ | 60ºC | [3] |
| *ENL* | Enl_for | 5’-CTTTGACTCGCGTGGCAAC-3’ | Enl_rev | 5’-CCTCCTCAATACGMAGAAGC-3’ | 60ºC | [3] |
| *HS90* | HSP90_F1  HSP90_F2 | 5’-GCTGGACACGGACAAGAACC-3’  5’-ATGGACAACTGCGAGGAGC-3’ | HSP90_R1  HSP90_R2 | 5’-ACACCCTTGACRAACGACAG-3’  5’-CGTGTCGTACAGCAGCCAGA-3’ | 62ºC | [3] |
| *ITS* | ITS4 | 5′-TCCTCCGCTTATTGATATGC-3′ | ITS6 | 5′-GAAGGTGAAGTCGTAACAAGG-3′ | 55ºC | [5] |
| *TigA* | Tig_for  G3PDH_for | 5’-TTCGTGGGCGGYAACTGG-3’  5’-TCGCYATCAACGGMTTCGG-3’ | Tig_rev  G3PDH_rev | 5’-CCGAAKCCGTTGATRGCGA-3’  5’-GCCCCACTCRTTGTCRTACCAC-3’ | 64ºC | [3] |

1. Moncalvo JM, Wang HH, Hseu RS. Phylogenetic relationships in *Ganoderma* inferred from the internal transcribed spacers and 25S ribosomal DNA sequences. Mycologia. 1995;87(2):223-38.

2. Riethmuller A, Voglmayr H, Goker M, Wei BM, Oberwinkler F. Phylogenetic relationships of the downy mildews (Peronosporales) and related groups based on nuclear large subunit ribosomal DNA sequences. Mycologia. 2002;94(5):834-49.

3. Blair JE, Coffey MD, Park SY, Geiser DM, Kang S. A multi-locus phylogeny for *Phytophthora* utilizing markers derived from complete genome sequences. Fungal Genetics and Biology. 2008;45(3):266-77.

4. Kroon L, Bakker F, Van Den Bosch G, Bonants P, Flier W. Phylogenetic analysis of *Phytophthora* species based on mitochondrial and nuclear DNA sequences. Fungal Genetics and Biology. 2004;41(8):766-82.

5. Cooke D, Drenth A, Duncan J, Wagels G, Brasier C. A molecular phylogeny of *Phytophthora* and related oomycetes. Fungal genetics and biology. 2000;30(1):17-32.
