# Supplementary material for: An open-access T-BAS phylogeny for emerging Phytophthora species
Source: PLoS One. 2023 Apr 3;18(4):e0283540. doi: 10.1371/journal.pone.0283540 (PMC10069789; doi:10.1371/journal.pone.0283540)
Supplement: S3 Table — (DOCX) [file pone.0283540.s010.docx]

**S3 Table**. Summary statistics for *Phytophthora infestans* SSR classifier by genotype class.

| Genotype | Number of Isolates | Sensitivity^a^ | Specificity^b^ | Prevalence^c^ | Detection Rate^d^ | Detection Prevalence^e^ | Balanced Accuracy^f^ |
| --- | --- | --- | --- | --- | --- | --- | --- |
| **North America** |  |  |  |  |  |  |  |
| US_1 | 62 | 1.0000 | 1.0000 | 0.0407 | 0.0407 | 0.0407 | 1.0000 |
| US_6 | 2 | 1.0000 | 1.0000 | 0.0013 | 0.0013 | 0.0013 | 1.0000 |
| US_7 | 4 | 1.0000 | 0.9987 | 0.0026 | 0.0026 | 0.0039 | 0.9993 |
| US_8 | 7 | 0.8571 | 0.9980 | 0.0046 | 0.0039 | 0.0059 | 0.9276 |
| US_11 | 2 | 0.5000 | 0.9993 | 0.0013 | 0.0007 | 0.0013 | 0.7497 |
| US_13 | 1 | 0.0000 | 0.9993 | 0.0007 | 0.0000 | 0.0007 | 0.4997 |
| US_14 | 1 | 0.0000 | 1.0000 | 0.0007 | 0.0000 | 0.0000 | 0.5000 |
| US_15 | 1 | 0.0000 | 0.9993 | 0.0007 | 0.0000 | 0.0007 | 0.4997 |
| US_16 | 1 | 0.0000 | 0.9993 | 0.0007 | 0.0000 | 0.0007 | 0.4997 |
| US_17 | 1 | 0.0000 | 1.0000 | 0.0007 | 0.0000 | 0.0000 | 0.5000 |
| US_18 | 3 | 1.0000 | 1.0000 | 0.0020 | 0.0020 | 0.0020 | 1.0000 |
| US_20 | 5 | 1.0000 | 1.0000 | 0.0033 | 0.0033 | 0.0033 | 1.0000 |
| US_21 | 2 | 1.0000 | 1.0000 | 0.0013 | 0.0013 | 0.0013 | 1.0000 |
| US_23 | 5 | 0.6000 | 1.0000 | 0.0033 | 0.0020 | 0.0020 | 0.8000 |
| US_25 | 1 | 0.0000 | 1.0000 | 0.0007 | 0.0000 | 0.0000 | 0.5000 |
| **Central and South America** |  |  |  |  |  |  |  |
| BR_1 | 5 | 1.0000 | 1.0000 | 0.0033 | 0.0033 | 0.0033 | 1.0000 |
| CR_1 | 18 | 0.9444 | 1.0000 | 0.0118 | 0.0111 | 0.0111 | 0.9722 |
| CR_2 | 3 | 0.3333 | 0.9993 | 0.0020 | 0.0007 | 0.0013 | 0.6663 |
| EC_1 | 786 | 1.0000 | 1.0000 | 0.5154 | 0.5154 | 0.5154 | 1.0000 |
| HN_1 | 12 | 0.7500 | 0.9974 | 0.0079 | 0.0059 | 0.0085 | 0.8737 |
| NI_1 | 22 | 1.0000 | 0.9993 | 0.0144 | 0.0144 | 0.0151 | 0.9997 |
| PE_3 | 81 | 1.0000 | 1.0000 | 0.0531 | 0.0531 | 0.0531 | 1.0000 |
| PE_7 | 40 | 0.9750 | 0.9993 | 0.0262 | 0.0256 | 0.0262 | 0.9872 |
| **Europe** |  |  |  |  |  |  |  |
| EU_1_A1 | 2 | 1.0000 | 1.0000 | 0.0013 | 0.0013 | 0.0013 | 1.0000 |
| EU_2_A1 | 21 | 0.9524 | 0.9993 | 0.0138 | 0.0131 | 0.0138 | 0.9759 |
| EU_12_A1 | 1 | 0.0000 | 1.0000 | 0.0007 | 0.0000 | 0.0000 | 0.5000 |
| EU_13_A2 | 295 | 0.9864 | 0.9976 | 0.1934 | 0.1908 | 0.1928 | 0.9920 |
| EU_23_A1 | 95 | 1.0000 | 0.9986 | 0.0623 | 0.0623 | 0.0636 | 0.9993 |
| EU_36_A2 | 2 | 1.0000 | 0.9993 | 0.0013 | 0.0013 | 0.0020 | 0.9997 |
| EU_8_A1 | 6 | 1.0000 | 0.9993 | 0.0039 | 0.0039 | 0.0046 | 0.9997 |
| FAM_1 | 27 | 1.0000 | 1.0000 | 0.0177 | 0.0177 | 0.0177 | 1.0000 |
| **Asia** |  |  |  |  |  |  |  |
| SIB_1 | 8 | 1.0000 | 1.0000 | 0.0052 | 0.0052 | 0.0052 | 1.0000 |
| CN_9 | 2 | 1.0000 | 1.0000 | 0.0013 | 0.0013 | 0.0013 | 1.0000 |
| CN_11 | 1 | 0.0000 | 1.0000 | 0.0007 | 0.0000 | 0.0000 | 0.5000 |

^a^true positive rate

^b^ true negative rate

^c^ the proportion of the dataset that is of this lineage

^d^ the rate at which the lineage was detected

^e^ the number of predicted positive lineages divided by the total number of lineages

^f^ arithmetic mean of sensitivity and specificity
