# Supplementary material for: An open-access T-BAS phylogeny for emerging Phytophthora species
Source: PLoS One. 2023 Apr 3;18(4):e0283540. doi: 10.1371/journal.pone.0283540 (PMC10069789; doi:10.1371/journal.pone.0283540)
Supplement: S1 File — (DOCX) [file pone.0283540.s012.docx]

T-BAS Public Tree Placement Instructions

Note: For placing taxa into the tree for species identification, we recommend sequencing at least three loci. The most informative loci for *Phytophthora* identification vary across the genus, so we recommend consulting “Differential Usefulness of Nine Commonly Used Genetic Markers for Identifying *Phytophthora* Species” by Yang and Hong (2018) to determine which loci are most applicable for species identification in your case.

1. Google “TBAS NCSU” and navigate to the [T-BAS webpage](https://vclv99-239.hpc.ncsu.edu/tbas2_3/pages/tbas.php).
2. To view the tree Select the “T-BAS Trees” option:


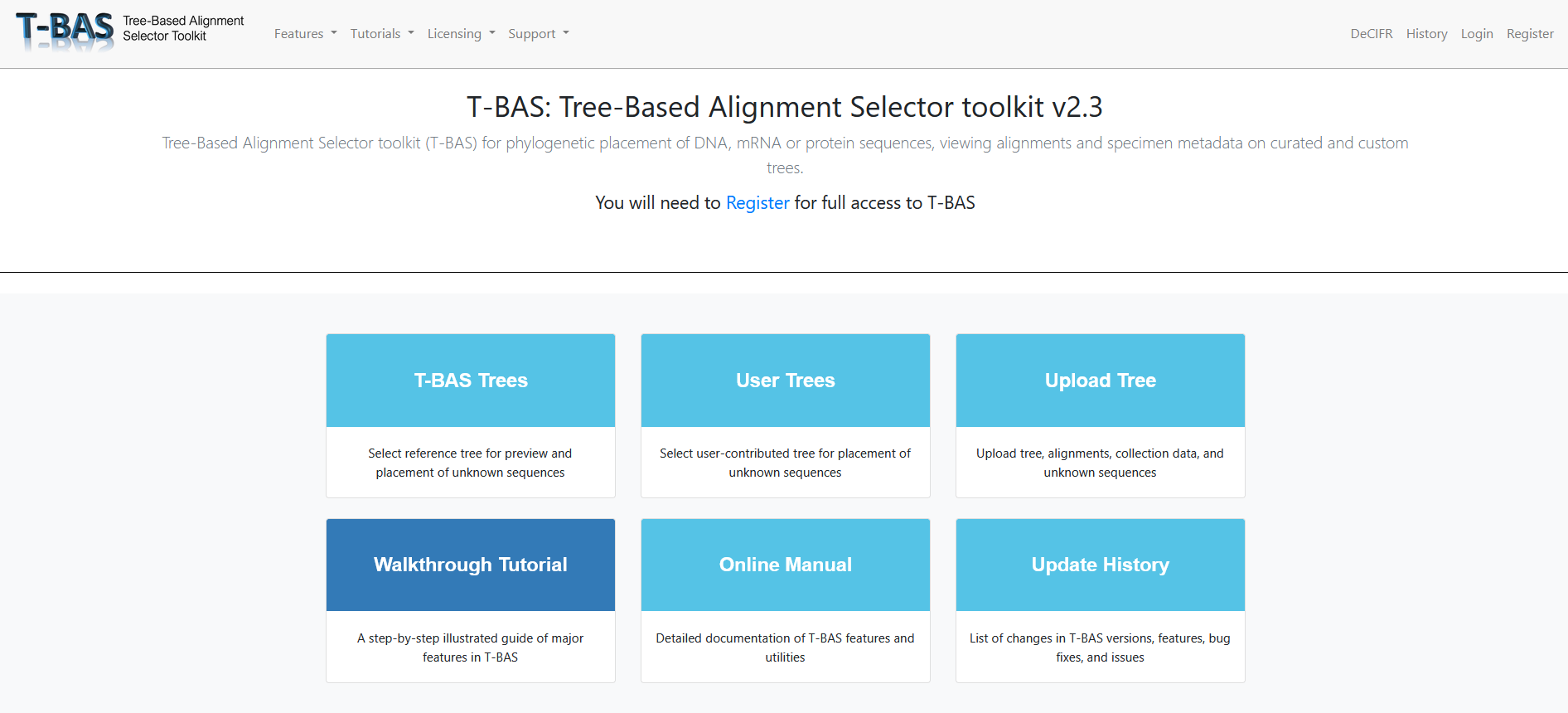


1. Select the **Phytophthora Nuclear** Tree from the tree of life. This is the recommended tree for taxa placement. The Phytophthora CoxI tree contains only the mitochondrial CoxI locus. Look at this if you are interested, but it is not recommended for placement.


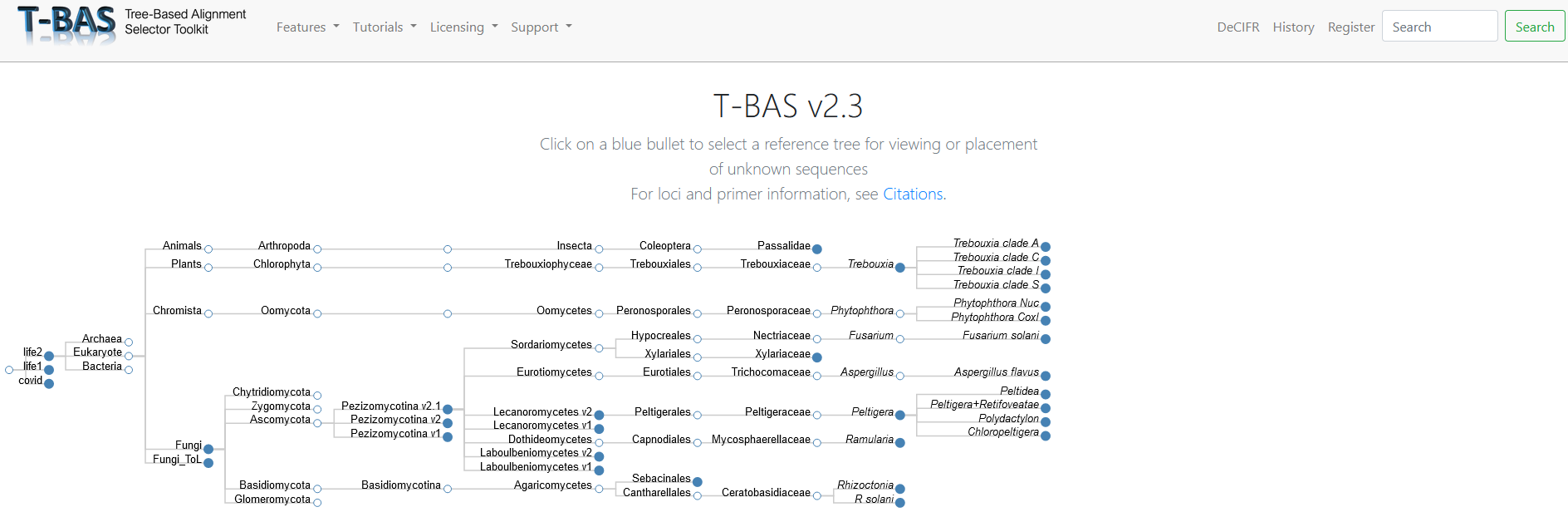


1. After selecting the tree, you should see two options. One for viewing the tree and one for placing taxa. Clicking view tree will open a new tab and load the tree, which takes a few moments. If you are interested in placing taxa, select “Place Unknowns”:


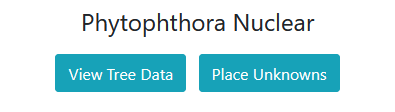


1. If you choose “Place Unknowns” a new screen will appear with several options. First, note the list of loci on the right (red box). These are the loci for which you can upload sequence data.
2. At the first input box, drag and drop sequence data in FASTA format for the taxa you would like to place.
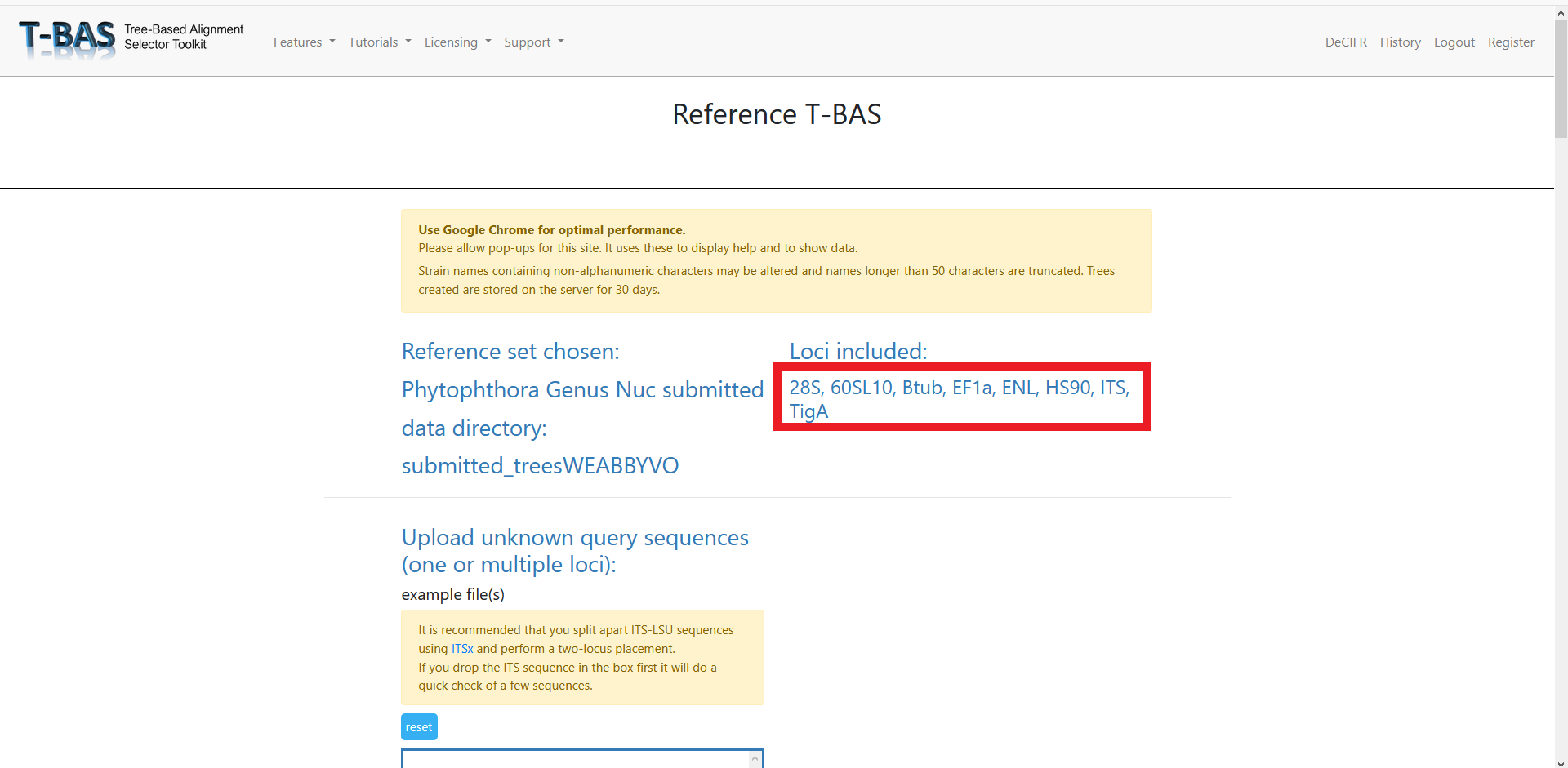
For this tutorial, example FASTA files have been provided in GitHub for each locus. They are named “locus_example.fasta” where ‘locus’ is the abbreviation for the relevant nuclear locus. Any or all of these files can be dragged and dropped into the Upload unknown query sequences box at this step.


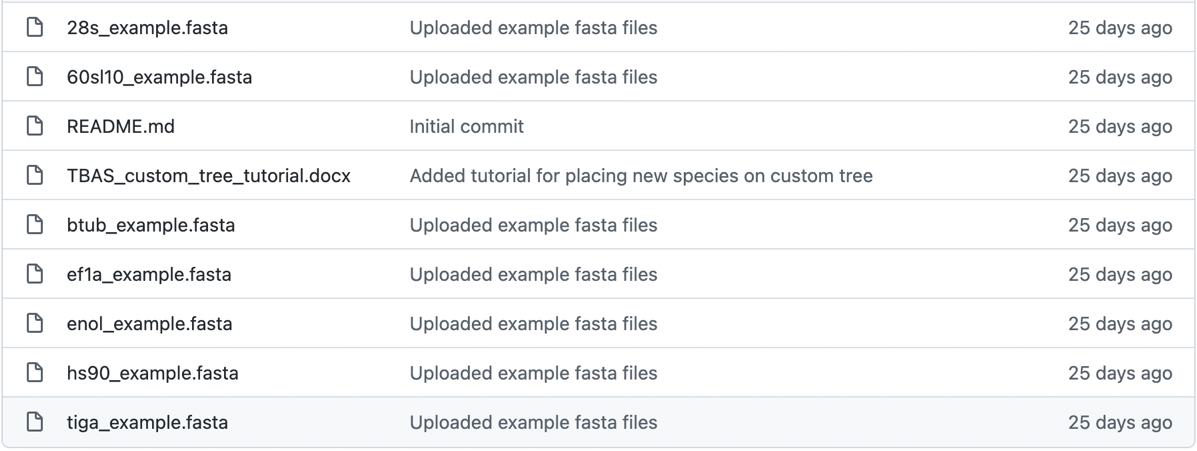

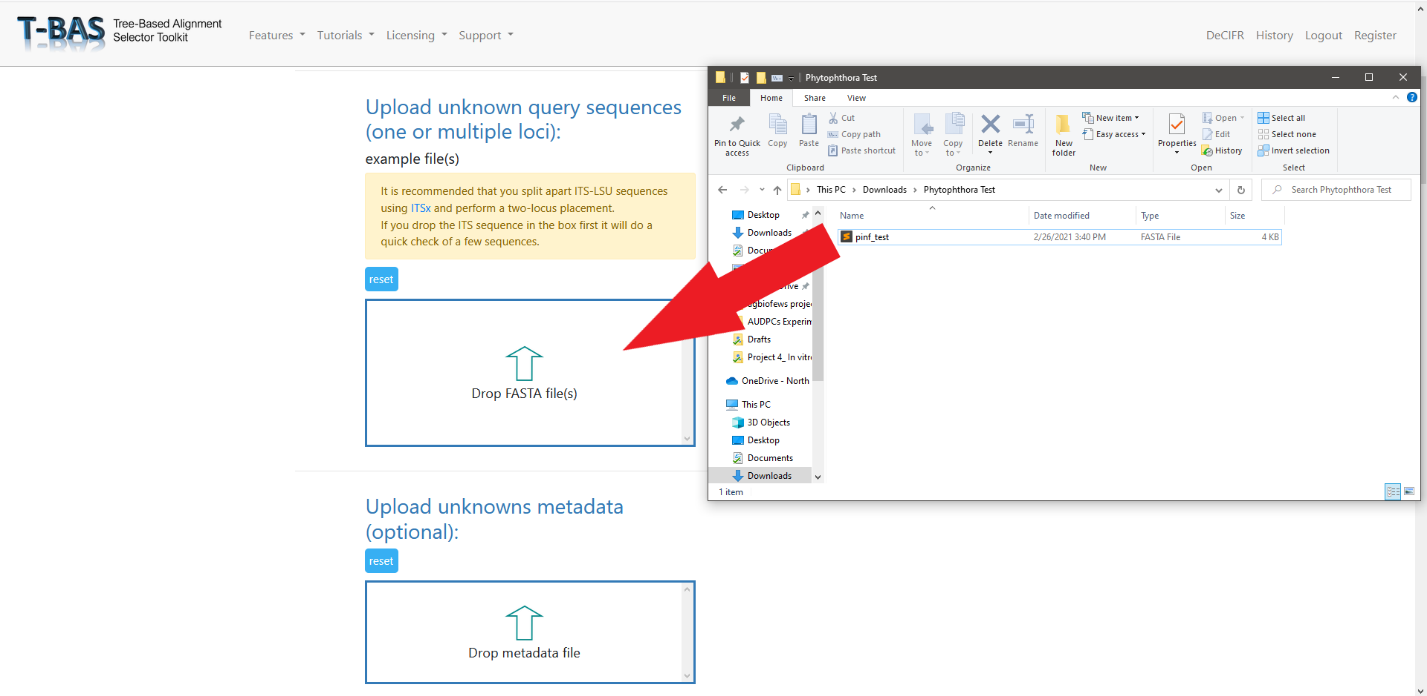


**Tips if you are uploading your own FASTA files:**

A separate FASTA file must be provided for each locus you are including.

If your FASTA files have multiple taxa, ensure the sequence headers for one taxon have the same name across files. For example, if you are uploading the HSP90 and TigA loci for two taxa named Phytophthora test1 and Phytophthora test2 your files should look like:


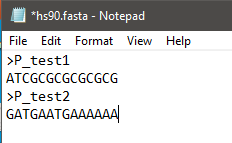

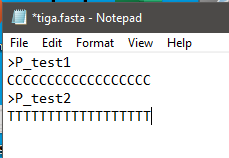


1.
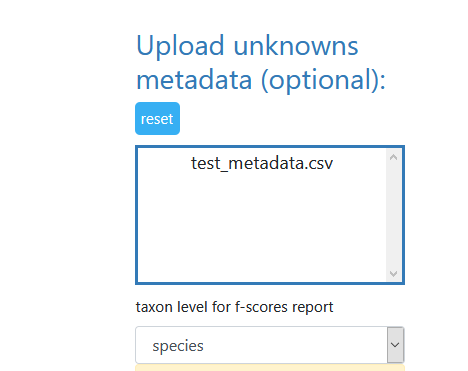
**Optional.** At the next dialog box, drag and drop a metadata file if you want to include one. If you do include a metadata file, change the drop-down menu below the dialog box from “Class” to “Species.” Metadata must be a CSV file, where the first column is your taxa names. An example of how to format the metadata, including the required headers, is available at the associated [GitHub](https://github.com/allisoncoomber/phytophthora_tbas) page.
2. Scroll down and click “Submit.” None of the other options need to be selected or changed. If you are interested in exploring phylogeny-based placement using a backbone constraint tree with bootstraps and other options, the TBAS [Tutorials](https://vclv99-239.hpc.ncsu.edu/tbas2_3/pages/tbas-tutorial.php) and [Manual](https://vclv99-239.hpc.ncsu.edu/tbas2_3/pages/tbas-documentation.php) can help you navigate them.
3. After you click submit, a new webpage will appear where you can select which loci your FASTA files correspond to. Choose from the drop-down menu, then click submit again.


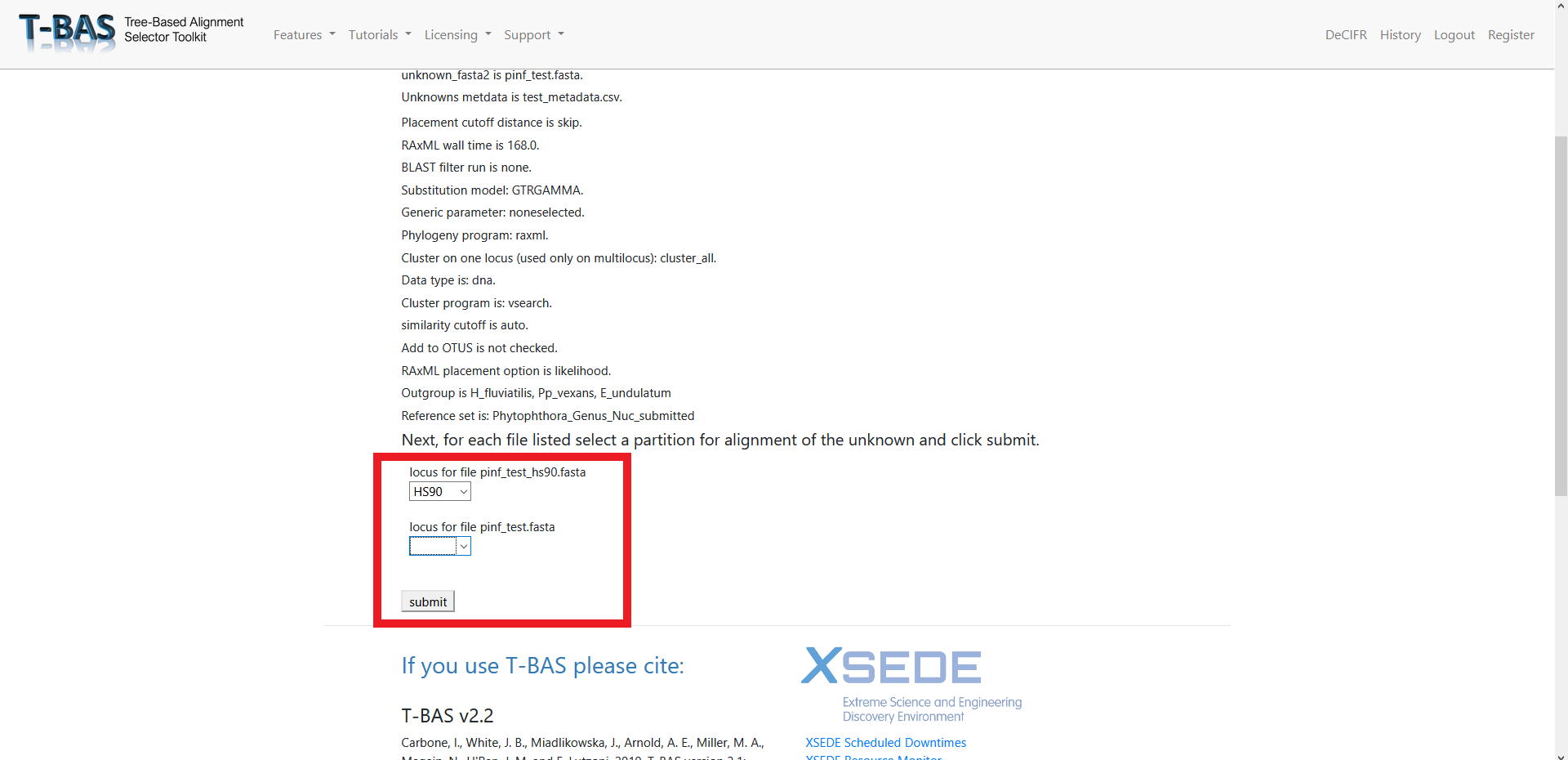


1. A loading bar will appear. Placement may take several minutes. When the tree placement is finished, several output files will be made available. Scroll down and click “View Tree.” This will open the tree in a new tab.


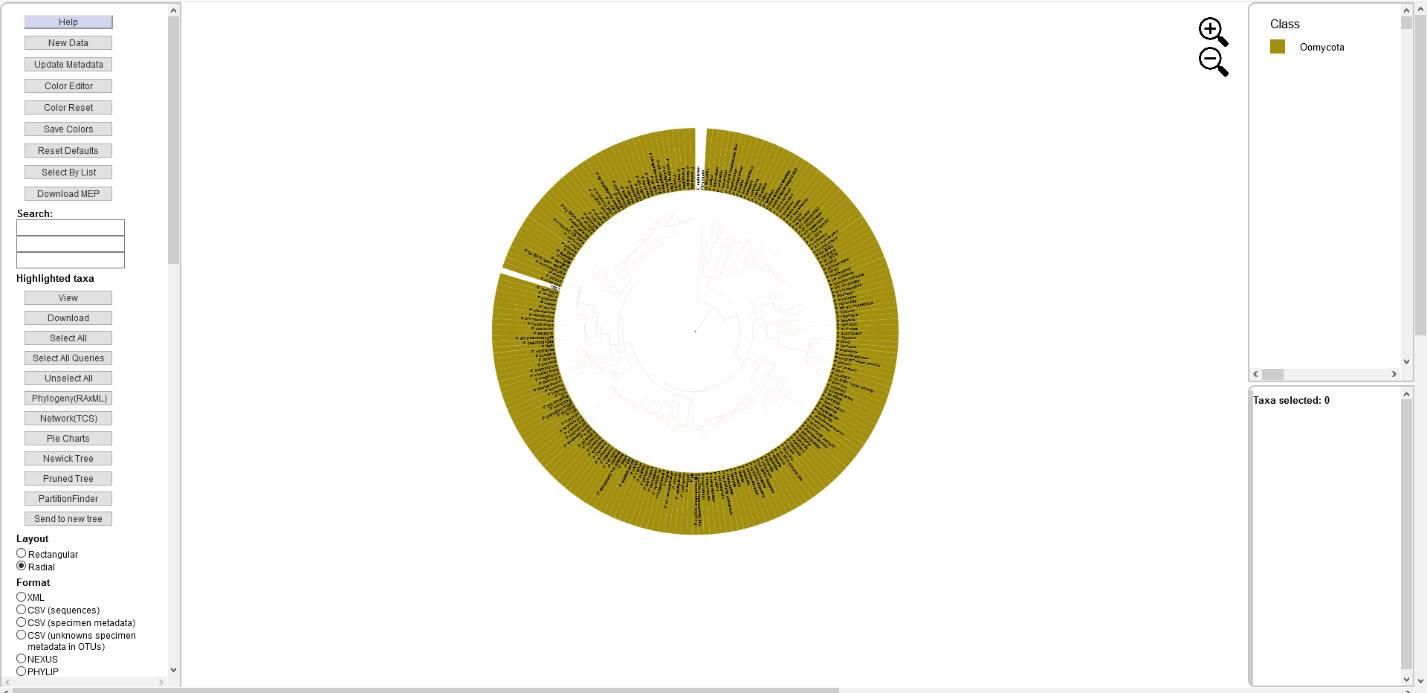


1. Select different options on the left as you choose to visualize the tree. We recommend colorizing leaves by different metadata, such as clade:


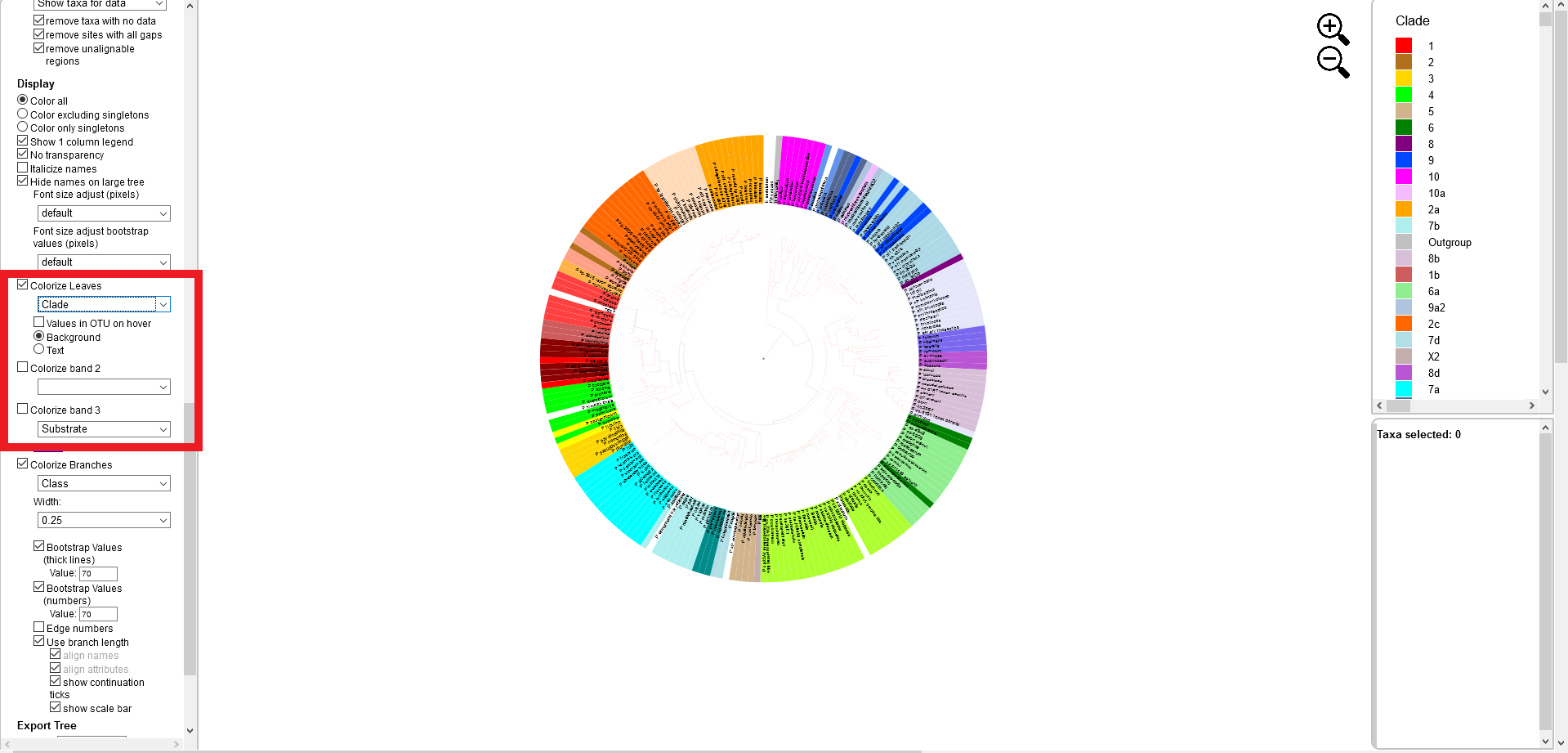


12. **Uploading your tree.** If you have made changes to the *Phytophthora* genus tree, such as adding a new species, and would like to add these changes into the reference tree on the NC State CIFR site, your new tree can be submitted. In the confirmation email you receive after placing your new taxa, click on the “submit to TBAS” button and follow the instruction to submit your new species for placement in the tree.

Citations:

Carbone, I., White, J. B., Miadlikowska, J., Arnold, A. E., Miller, M. A., Kauff, F., U'Ren, J. M., May, G. and F. Lutzoni. 2017. T-BAS: Tree-Based Alignment Selector toolkit for phylogenetic-based placement, alignment downloads, and metadata visualization; an example with the Pezizomycotina tree of life. Bioinformatics 33: 1160-1168. DOI: 10.1093/bioinformatics/btw808

Carbone, I., White, J. B., Miadlikowska, J., Arnold, A. E., Miller, M. A., Magain, N., U'Ren, J. M. and F. Lutzoni. 2019. T-BAS version 2.1: Tree-Based Alignment Selector toolkit for evolutionary placement of DNA sequences and viewing alignments and specimen metadata on curated and custom trees. Microbiology Resource Announcements Microbiol Resour Announc 8:e00328-19. https://doi.org/10.1128/MRA.00328-19.

T-BAS v2.3 <https://vclv99-239.hpc.ncsu.edu/tbas2_3/pages/tbas.php>

Yang, X., & Hong, C. (2018). Differential usefulness of nine commonly used genetic markers for identifying *Phytophthora* species. Frontiers in microbiology, 9, 2334.
