# Supplementary material for: An open-access T-BAS phylogeny for emerging Phytophthora species
Source: PLoS One. 2023 Apr 3;18(4):e0283540. doi: 10.1371/journal.pone.0283540 (PMC10069789; doi:10.1371/journal.pone.0283540)
Supplement: S2 File — (DOCX) [file pone.0283540.s013.docx]

**Supplemental File 1. SSR Classifier Tutorial**

This program identifies unknown lineages of *Phytophthora infestans* based on 12 commonly used SSR markers (microsatellite data). The program is hosted on the [USABlight.org](usablight.org) website under “[Identify SSR Genotype](https://usablight.org/identify-ssr-genotype/)” and the database represents most of the currently named SSR lineages of *P. infestans*.

**How it works**

The SSR classifier compares the SSR data for your isolates of interest to over 2,500 described isolates from global populations in our *P. infestans* database. The closest genetic match is determined using Bruvo’s distance (described [here](https://doi.org/10.1111/j.1365-294x.2004.02209.x)).

**How to run SSRs**

This program uses results obtained from a 12-plex microsatellite amplification for *Phytophthora infestans*. These 12 microsatellite loci are well described for *Phytophthora infestans* and vary across lineages, allowing identification of different genotypes of the pathogen. Genotyping using this SSR method first involves determining the microsatellite alleles for each of the 12 loci. The methods for running SSRs have been previously described [here](https://doi.org/10.1016/j.mimet.2012.11.021). A simplified version of the protocol can be found on the USABlight.org website under “SSR Genotyping Methods.”

**How to format data**

The raw microsatellite reads obtained from SSR genotyping must be processed and scored to create input data for the SSR classifier. Once the alleles are determined from the peak data, they can be formatted. Use the [SSR matcher sample submission template CSV file](https://usablight.org/identify-ssr-genotype/) found on USABlight.org and upload to this tool for genotype identification. Three numbers are separated by forward slashes for each locus to represent all three alleles in triploid individuals. If only two alleles are present, the third number is a zero. There is one column for each locus, along with metadata columns for sample name, genotype, region, country, date, host, number of missing loci, and number of present loci. Each row beneath the header represents the data for one sample.


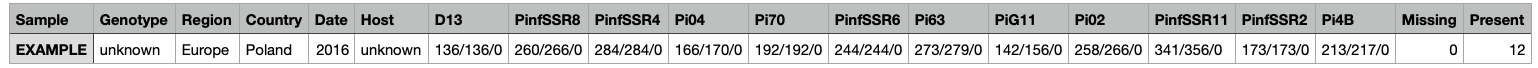


**Fig. 1**. Example formatting of an input CSV for the SSR classifier.

**How to upload data**

Upload a CSV file containing metadata about your sample as well as SSR data for the 12 loci of interest. Follow the format in the [SSR matcher sample submission template CSV file](https://usablight.org/identify-ssr-genotype/). We recommend downloading this CSV, replacing the data with your own, and re-uploading it. The classifier will still work if your data is missing some loci, but at least 10 loci are recommended for the most accurate results. Include as many samples as you want. The user can upload multiple lineages into the CSV file with appropriate metadata linked.

**How to interpret results**

If your unknown sample has a close match, the program will return information about the match from our database of *Phytophthora infestans* genotypes (**Fig. 2**). The tool first presents a table of input samples with their closest match and metadata associated with the closest match. For example, in Figure 2, input sample EG_256 most closely matches an isolate of lineage EU_23_A1 found in Egypt. After this table follows a more in-depth description of each match and where to learn more about the matching genotype. Finally, a list of all submitted samples for which no match was found are listed. Note just because it shows a match to an Egyptian genotype, this does not mean that was the source. The region, country, sample date and host are additional metadata from the isolate we used in developing the classifier and can be used in downstream analysis.

**
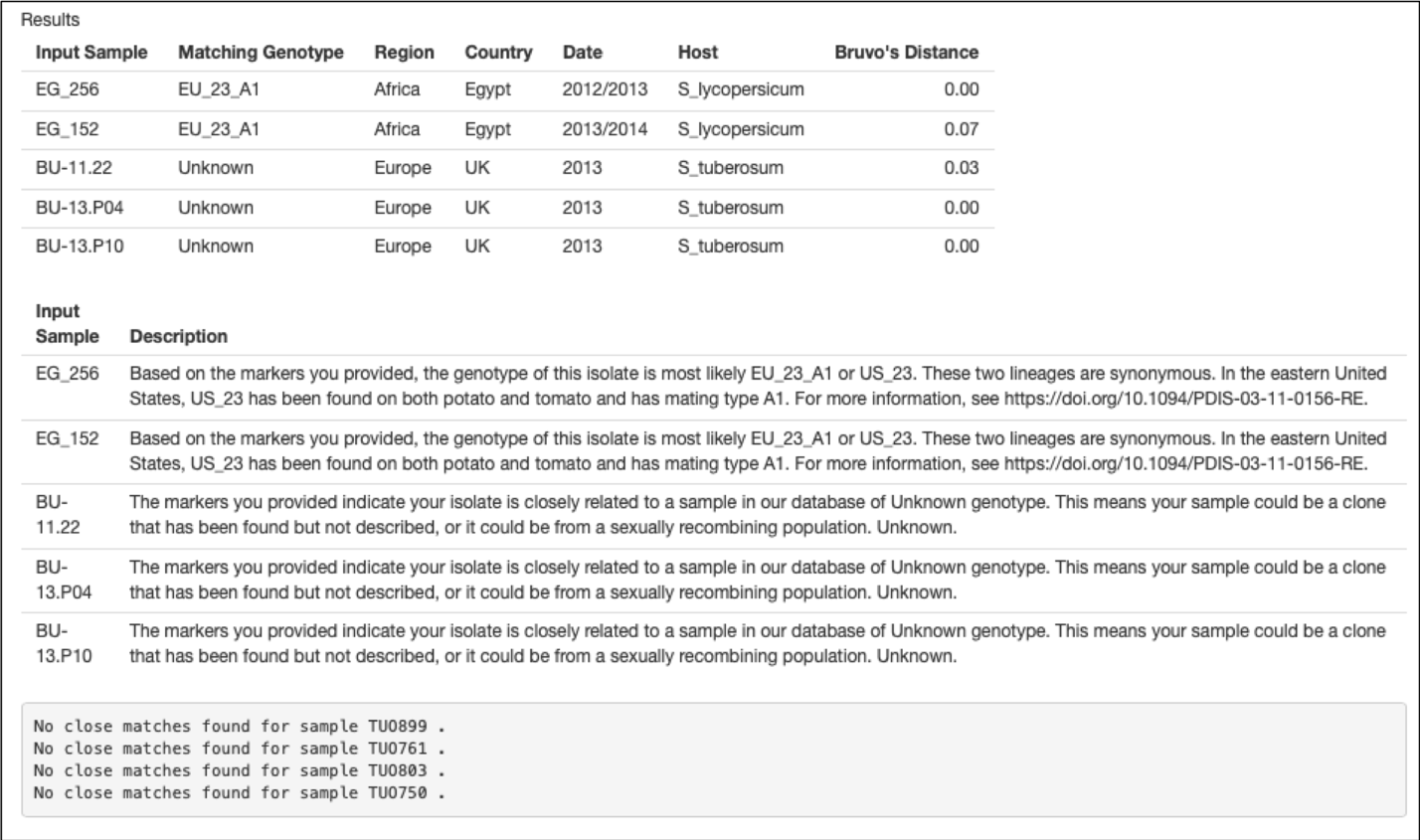
**

**Figure 2**. Example output from SSR classifier.

**Having issues?**

If you are unable to upload your samples and run the SSR classifier, please email the CSV file you are using and the problem occurring to [acoombe@ncsu.edu](mailto:acoombe@ncsu.edu) or [acsavill@ncsu.edu](mailto:acsavill@ncsu.edu).

**References**

Li Y, Cooke DE, Jacobsen E, van der Lee T. Efficient multiplex simple sequence repeat genotyping of the oomycete plant pathogen *Phytophthora infestans*. Journal of microbiological methods. 2013;92(3):316-22.

Saville A, Ristaino, JB. Genetic structure and subclonal variation of extant and recent US lineages of *Phytophthora infestans*. Phytopathology. 2019;109(9):1614-27.

Saville, A.C., La Spada, F., Faedda, R, Migheli, Q., Scanu, B, Ermacora, et al.  Population structure of Phytophthora infestans in Italy, Algeria and Tunisia. Plant Pathol. 2021; 7: 2165-2178.

**Other Resources**

[USABlight](https://usablight.org/): Genotype tracking website for North America

[EuroBlight](https://agro.au.dk/forskning/internationale-platforme/euroblight/): Genotype tracking website for Europe

[Tizón Latino](https://tizonlatino.github.io/): Genotype tracking website for Latin America

[Help notes for scoring SSR alleles in *Phytophthora infestans*](https://agro.au.dk/fileadmin/euroblight/Protocols/Pinf_SSR_scoring_Guide_DCooke_May2019.pdf): Allele calling guide developed by David Cooke.

For information on the SSR binning scheme used by this tool, see “[Population structure of *Phytophthora infestans* collected on potato and tomato in Italy](https://doi.org/10.1111/ppa.13444)” by Saville et al. 2021, Supplemental Table 3.
